# Microbially mediated fossil concretions and their characterization by the latest methodologies: a review

**DOI:** 10.3389/fmicb.2023.1225411

**Published:** 2023-09-29

**Authors:** Navdeep K. Dhami, Paul F. Greenwood, Stephen F. Poropat, Madison Tripp, Amy Elson, Hridya Vijay, Luke Brosnan, Alex I. Holman, Matthew Campbell, Peter Hopper, Lisa Smith, Andrew Jian, Kliti Grice

**Affiliations:** ^1^Western Australian – Organic and Isotope Geochemistry Centre (WA-OIGC), School of Earth and Planetary Sciences, The Institute for Geoscience Research, Curtin University, Perth, WA, Australia; ^2^The Trace and Environmental DNA lab (trEND), School of Molecular and Life Sciences, Curtin University, Perth, WA, Australia

**Keywords:** concretion, fossil, microbes, organic geochemistry, paleontology, biomarkers, biomolecules, biominerals

## Abstract

The study of well-preserved organic matter (OM) within mineral concretions has provided key insights into depositional and environmental conditions in deep time. Concretions of varied compositions, including carbonate, phosphate, and iron-based minerals, have been found to host exceptionally preserved fossils. Organic geochemical characterization of concretion-encapsulated OM promises valuable new information of fossil preservation, paleoenvironments, and even direct taxonomic information to further illuminate the evolutionary dynamics of our planet and its biota. Full exploitation of this largely untapped geochemical archive, however, requires a sophisticated understanding of the prevalence, formation controls and OM sequestration properties of mineral concretions. Past research has led to the proposal of different models of concretion formation and OM preservation. Nevertheless, the formation mechanisms and controls on OM preservation in concretions remain poorly understood. Here we provide a detailed review of the main types of concretions and formation pathways with a focus on the role of microbes and their metabolic activities. In addition, we provide a comprehensive account of organic geochemical, and complimentary inorganic geochemical, morphological, microbial and paleontological, analytical methods, including recent advancements, relevant to the characterization of concretions and sequestered OM. The application and outcome of several early organic geochemical studies of concretion-impregnated OM are included to demonstrate how this underexploited geo-biological record can provide new insights into the Earth’s evolutionary record. This paper also attempts to shed light on the current status of this research and major challenges that lie ahead in the further application of geo-paleo-microbial and organic geochemical research of concretions and their host fossils. Recent efforts to bridge the knowledge and communication gaps in this multidisciplinary research area are also discussed, with particular emphasis on research with significance for interpreting the molecular record in extraordinarily preserved fossils.

## Introduction

The fossil record is fundamental to our understanding of major events in the evolution of life and our planet. However, the fossil record suffers from numerous biases, and not all fossils are created equally (Allison and Bottjer, 2011). The process of fossilization is a complex interplay of biological, microbial and geochemical processes including decay and preservation through mineralization (Briggs, 2003b).

**Figure 1:**
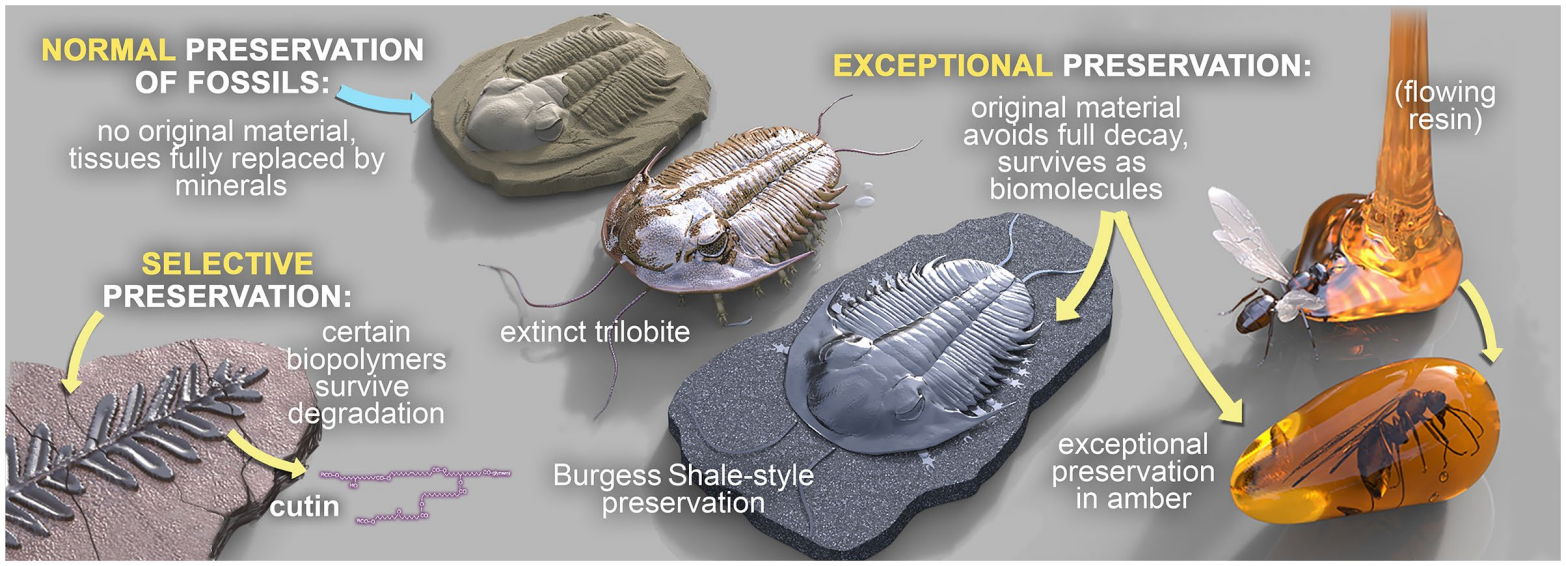
The three broad modes of fossilization: ‘Normal’ fossilization, ‘Selective’ fossilization, and ‘Exceptional’ fossilization. Fossils in concretions can be preserved in any of these modes, but those that are exceptionally preserved are of particular interest.

Three broad modes of fossilization have been proposed previously (Grice et al., 2019), as summarized in Figure 1. In **‘normal’ fossil preservation**, the remains of dead organisms are subject to a range of complex biogeochemical processes encompassing degradation via eogenesis and diagenesis (within the water column and surface sediments), burial, microbial activity, authigenic mineralization and metagenesis (in sediments), weathering and exhumation. These processes destroy nearly all OM. In **‘selective’ fossil preservation**, certain extremely resistant or recalcitrant biopolymers are fossilized. These include, for example, leaf cuticles of higher plants (Nip et al., 1986; Goth et al., 1988; Gelin et al., 1994; Grice et al., 2001, 2003) and the cell walls of marine and freshwater algae, which are relatively persistent in geological sediments. High molecular-weight (MW), aliphatic biomolecules are some of the more recalcitrant organic compounds (Tissot and Welte, 1984). Finally, a small fraction of organisms die in environments conducive to soft tissue preservation, which is termed **‘exceptional’ fossil preservation**. Exceptionally preserved fossils have the potential to preserve biomarkers, and in special cases intact biolipids (e.g., sterols), which retain important information about extinct organisms and are vital for establishing paleodiets and reconstructing paleoenvironments at the microbial level (Grice et al., 2019; Tripp et al., 2022).

One fossilization pathway that appears to have high potential for exceptional fossilization, and therefore for soft tissue preservation, is concretion formation (Irwin et al., 1977; Martill, 1990; Briggs and Kear, 1993a,c; Bojanowski and Clarkson, 2012; McCoy, 2014; Cotroneo et al., 2016). When this process is sufficiently rapid, it decreases permeability and consequently protects OM from significant degradation, allowing ‘**closed chemical system’** preservation of soft tissues (Allison and Pye, 1994; Farmer and Des Marais, 1999; McCoy et al., 2015b). This is a relatively uncommon pathway, but has led to the soft parts of various organisms being fossilized and entombed in concretions across a vast spatiotemporal range (Martill, 1990, 1993; Trinajstic et al., 2007, 2022a,b; Marshall and Pirrie, 2013; McCoy, 2014). Concretionary lagerstätten are known to represent a variety of different palaeoenvironments, from hypersaline to marine, estuarine to lacustrine, and freshwater to terrestrial (Marshall and Pirrie, 2013; McCoy, 2014), but the majority appear to pertain to transitional environments, i.e., those at the interface between the marine and terrestrial realms (McCoy et al., 2015a). Whole or parts of organisms can be preserved via several processes, including entombment or primary cementation in concretions of calcite, silica, phosphate, siderite, pyrite or dolomite, with carbonate concretions being the most common (Baird et al., 1986; Allison and Pye, 1994; McCoy, 2014). Concretions are often hosted in fine-grained sedimentary rocks like shales and mudstones (McCoy, 2014). Their relatively low permeability can effectively implement a closed chemical system, isolating the remains of the organism from other organic matter and redox influences thereby inhibiting diagenetic reactions and supporting the preservation of fossilized soft tissue (s) and biosignatures (McCoy et al., 2015a,b). Although the size of the nucleating fossil within the concretion is often the controlling factor on concretion size, this is not always the case: for example, sub-centimeter ostracods form the nuclei of multi-meter concretions in the Devonian-aged Huron Member of the Ohio Shale (Clifton, 1957).

Recently, it has been hypothesized from fossil evidence that microbial metabolic processes are responsible for the preservation and mineralization of the organic tissues in the fossil record (Briggs, 1995b, 2003a,b; Sagemann et al., 1999; O'Brien et al., 2002; Briggs et al., 2005; Raff et al., 2008). Microorganisms live both on the surface of, and within, living organisms, and they can remain active on or in a carcass well after the time of death of an organism (Allison, 1988a; Briggs, 1995b; Sagemann et al., 1999; Janssen et al., 2022). Certain microbially-induced carbonate concretions have recently been recognized for their excellent potential to sequester OM, including organic compounds preserving a direct link to their biological origins (i.e., molecular fossils or biomarkers) and often also providing valuable information about past depositional environments (Melendez et al., 2013a,b; Plet et al., 2016). Initial heterotrophic decay by microorganisms leads to the release of high concentrations of ions ranging from H^+^, OH^−^, Na^+^, K^+^, Ca^2+^, Mg^2+^, Fe^3+^, NH^4+^, S^2−^ into their immediate environment. This can facilitate the stabilization of the remaining tissues of a carcass and can influence mineralization; thus, microbial activity can lead to excellent preservation, provided that the rate of mineralization or stabilization exceeds that of tissue decomposition (Gehling, 1999; Sagemann et al., 1999; Petrovich, 2001; Carpenter, 2005; Janssen et al., 2022). Theoretical models and experimental observations that have highlighted the role of microbes in the fossilization process have also identified the physico-chemical conditions that can inhibit microbial mineral precipitation (Gehling, 1999; Briggs, 2003a; Martin et al., 2003; Bolhuis et al., 2013; Gäb et al., 2020). The influence of microbial activities on fossilization processes occurs in the early stages of eogenesis and diagenesis (Dupraz and Visscher, 2005; Raff et al., 2013; Dang and Lovell, 2016; Janssen et al., 2022). Detailed investigations into the role of intrinsic and extrinsic microbial metabolic activities, prevalence of sites/types of fossils, role of substrate, formation controls, bio-physico-chemical environments, type of organic matrix, governing reaction kinetics and comprehensive characterization of biomolecules from nano- to micro-scales are pivotal in understanding the fossilization story over geologic timeframes.

This review discusses the study of well-preserved OM in mineral concretions and how it can provide information on depositional and environmental conditions and the evolutionary history of life on Earth. Various concretion types and their formation modes are described, as well as the variety of methodologies employed in their characterization. The review also includes examples of organic geochemical (biomarker) and isotopic studies and the enormous potential for the molecular record to reveal insights about extinct organisms. Challenges and knowledge gaps in the field are presented, along with recent efforts to bridge them.

## Exceptionally preserved fossils in concretions through space and time

**Figure 2:**
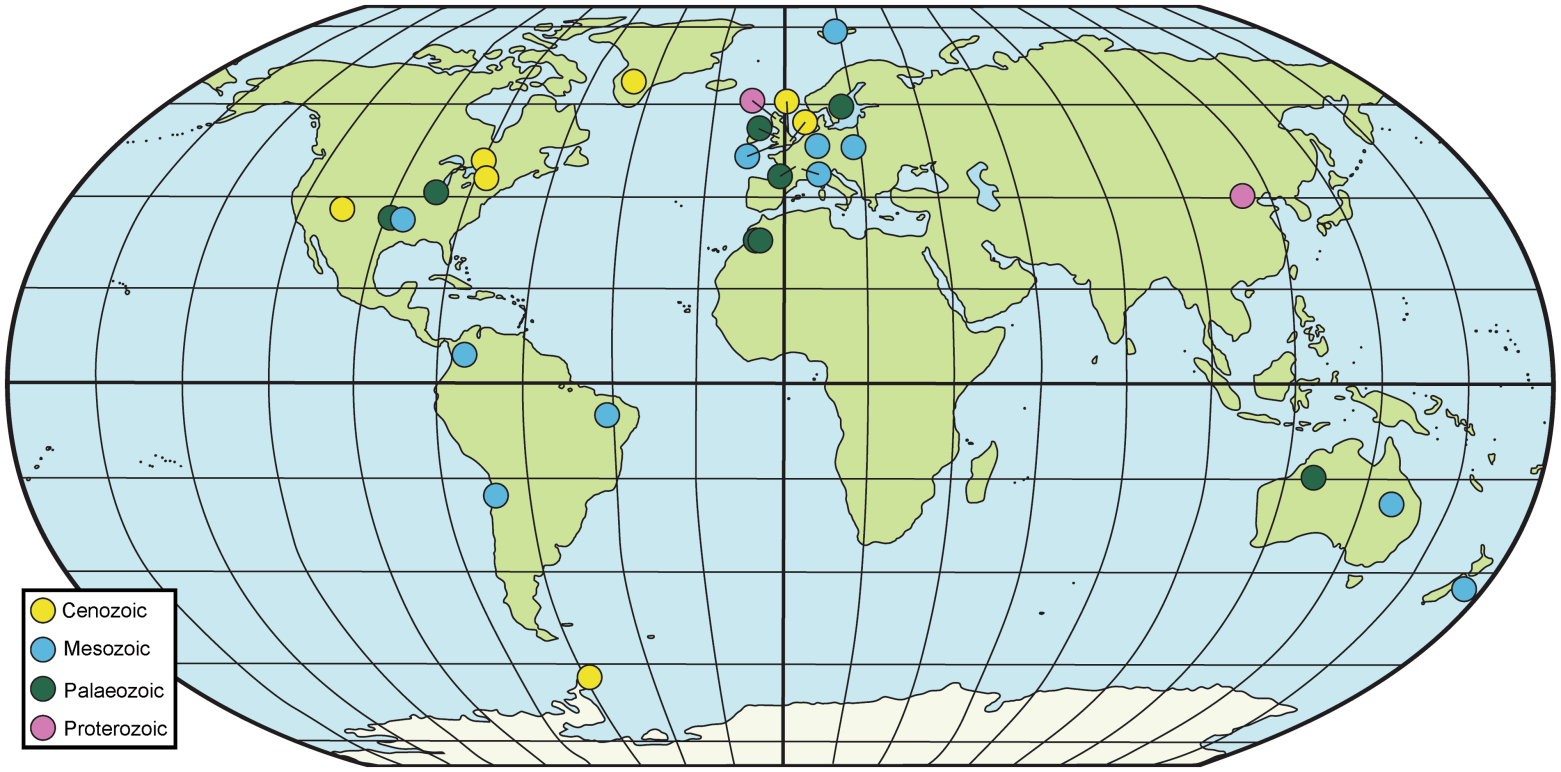
Map of selected geological sites hosting fossiliferous concretions. Particularly prominent sites are summarized in Table 1, although others mentioned in the text are also included here.

**Figure 3:**
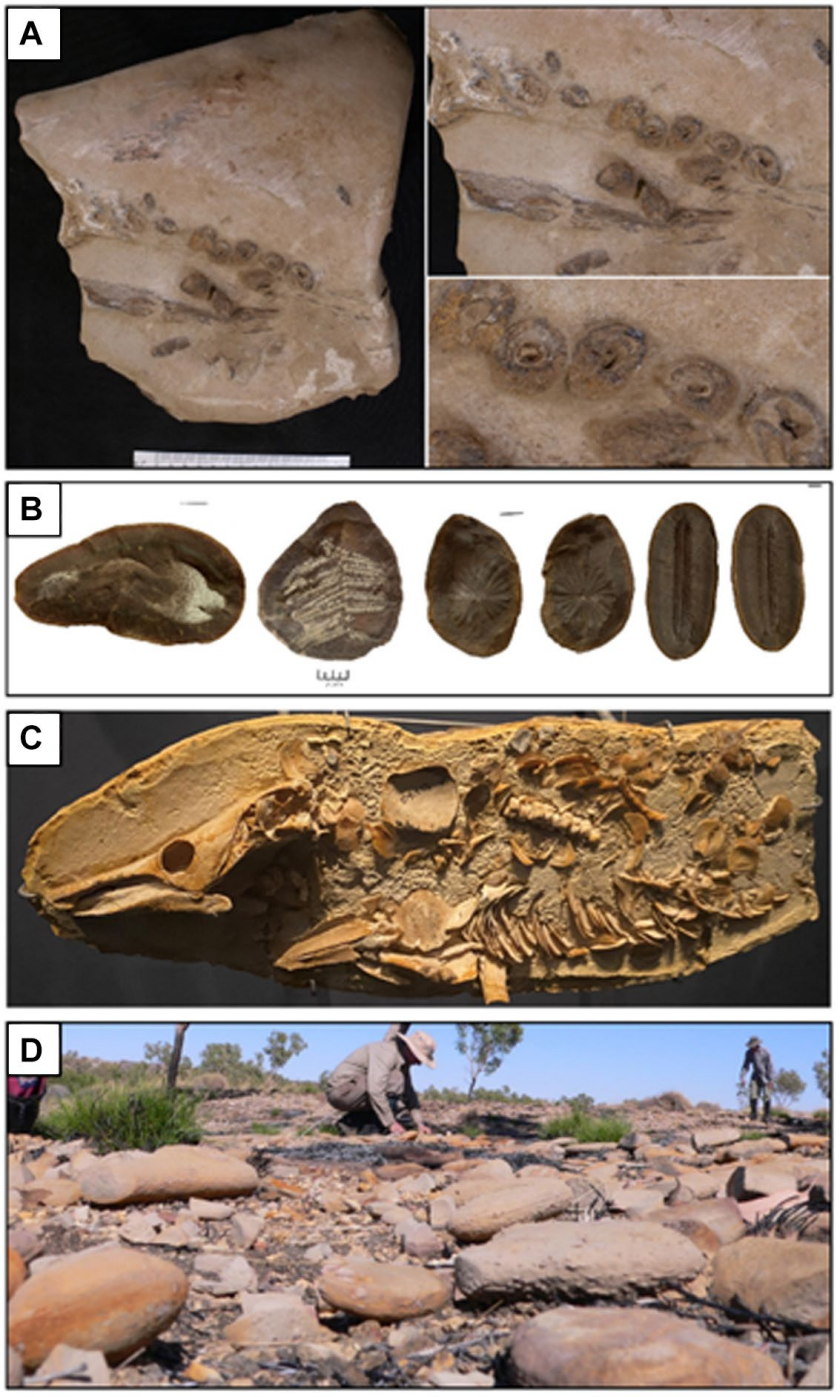
Concretions from various sites that host exceptionally preserved fossils. **(A)** Ichthyosaur [cf. *Platypterygius australis* (McCoy)] jaw and teeth preserved within a concretion from the upper Albian Toolebuc Formation of Richmond, Queensland, Australia. **(B)** Several fossils from the Upper Carboniferous Francis Creek Shale Member of the Carbondale Formation of Mazon Creek, Illinois, USA. From left–right: the enigmatic animal *Tullimonstrum gregarium* Richardson; the fern *Pecopteris* sp.; the horsetail *Annularia steelata*; and the fern *Diplozites unita*. **(C)** Partially prepared skeleton of the lungfish *Griphognathus whitei* from the Middle–Upper Devonian Gogo Formation of the Canning Basin, Western Australia (photograph A. M. Clement). **(D)** Concretions in the field being inspected during fieldwork focused on the Middle–Upper Devonian Gogo Formation, Canning Basin, Western Australia (photograph J. A. Long).

Fossiliferous concretions have been identified in strata ranging in age from Mesoproterozoic to Holocene (Mozley and Burns, 1993; Marshall and Pirrie, 2013; McCoy, 2014; Wang et al., 2017; Mojarro et al., 2022) as seen in Figures 2, 3 and Table 1. The Mesoproterozoic Xiamaling Formation (~1.37 giga annum [Ga]), which preserves silicified bitumen concretions in black shale and green chert, antedates the evolution of multicellular organisms. Consequently, the nuclei for these concretions are bacterial and include microbial mats (Wang et al., 2017; Liu et al., 2019; Liu A. et al., 2020). Concretion-bearing deposits are far more common in Phanerozoic deposits than in Proterozoic or older strata. In lower Paleozoic deposits, the fossils dominantly preserved in concretions are invertebrates; examples of deposits where this is the case are the upper Cambrian Alum Shale Formation of Sweden (Dworatzek, 1987; Maeda et al., 2011) and the lower Ordovician Fezouata Formation of Morocco (Gaines et al., 2012; Van Roy et al., 2015; Saleh et al., 2021). The rocks hosting the concretions in each of these cases are shales, but the concretions in the former are calcareous, whereas those in the latter are siliceous. The middle Silurian Coalbrookdale Formation of the UK (Herefordshire Lagerstätte) also preserves invertebrates with soft tissue in calcareous concretions, but the host rock in this instance is bentonite (Siveter et al., 2020).

**Table 1:** A selection of sites preserving fossiliferous concretions.

Locality	Formation	Age	Preservation	Fossils present	References
Norfolk, United Kingdom	-	Holocene	Siderite, calcite and iron monosulfide concretions	Plants, Invertebrates	Pye (1984), Pye et al. (1990), Allison and Pye (1994)
Kangerlussuaq, Greenland	-	Holocene	Carbonate concretions in clay	Fish (capelin: *Mallotus villosus*)	Mojarro et al. (2022)
Ottawa, Canada	-	Holocene	Carbonate concretions in clay	Fish (capelin: *Mallotus villosus*)	Mojarro et al. (2022)
Kent, United Kingdom	London Clay Formation	lower Eocene	Pyrite, apatite, phosphate, and calcite concretions in clay	Plants, Invertebrates, Fishes, Reptiles, Birds, Mammals	Allison (1988b), Huggett (1994), Huggett et al. (2000)
Magdalena Valley, Colombia	Cretaceous black shales	Lower Cretaceous	Carbonate concretions in shales	Invertebrates, Fishes	Weeks (1953, 1957)
Santana Group, Chapada do Araripe, Brazil	Romualdo Formation, Santana Group	Lower Cretaceous	Carbonate concretions in mudstones/shales with minor limestone	Plants, Invertebrates, Fishes, Amphibians, Reptiles, Birds	Mabesoone and Tinoco (1973), Martill (1988, 1989, 1990, 1993, 2007), Maisey (1991), Fara et al. (2005), Varejão et al. (2021)
Cerritos Bayos, Chile	Cordillera de Domeyko	Upper Jurassic	Calcareous concretions in black, sandy, gypsum-rich shales	Invertebrates, Fishes, Reptiles	Schultze (1989), Arratia and Schultze (1999)
Posidonia Shale, Germany	Sachrang Formation	Lower Jurassic	Pyritiferous calcite concretions in shale	Plants, Invertebrates, Fishes, Reptiles	Plet et al. (2016, 2017)
Mazon Creek, Ilinois, USA	Francis Creek Shale Member, Carbondale Formation	Upper Carboniferous	Siderite (iron carbonate) concretions in shale	Plants, Invertebrates, Fishes, Amphibians, Reptiles	Johnson and Richardson (1966), Woodland and Stenstrom (1979), Baird et al. (1985a, 1985b, 1986), Archer et al. (1995), Clements et al. (2018)
Gogo Station, Western Australia, Australia	Gogo Formation	Middle–Upper Devonian	Calcareous concretions hosted in shales and siltstones with lenses of limestone	Radiolarians, Invertebrates, Fishes	Melendez et al. (2013b), Lengger et al. (2017), Trinajstic et al. (2022a,b)
Herefordshire, United Kingdom	Coalbrookdale Formation	middle Silurian	Carbonate concretions hosted in bentonite	Invertebrates	Siveter et al. (2020)
Fezouata Biota, Morocco	Fezouata Formation	Lower Ordovician	Siliceous concretions in shale	Invertebrates (anomalocaridids)	Gaines et al. (2012), Van Roy et al. (2015), Saleh et al. (2021)
Västergötland, Sweden	Alum Shale Formation	upper Cambrian	Carbonate concretions in black shale (‘Orsten’-style concretions)	Invertebrates	Dworatzek (1987), Maeda et al. (2011)
North China	Xiamaling Formation	middle Mesoproterozoic (~1.39 Ga)	Silicified bitumen concretions in black shale, green chert	Bacteria; biofilms	Wang et al. (2017), Liu et al. (2019), Liu A. et al. (2020)

Note the broad geographic and temporal ranges of these deposits. Whether or not the concretions at these sites (or others not included in this table) were formed as a consequence of microbial activity remains to be demonstrated.

One of the most famous concretion-bearing and soft-tissue preserving fossil deposits in the world is the Middle–Upper Devonian Gogo Formation of Western Australia (Long and Trinajstic, 2010; Melendez et al., 2013b; Lengger et al., 2017; Trinajstic et al., 2022a). Fossils from this unit have provided unprecedented insights into the soft tissue anatomy and ecology of early fishes (e.g., Trinajstic et al., 2007, 2013, 2022b; Long et al., 2008, 2009; Ahlberg et al., 2009). In addition, molecular and isotopic studies of a fossil crustacean from this unit revealed the oldest intact biolipids (i.e., cholesterol) in the fossil record, as well as the entire diagenetic continuum of steroids representing transformations that occurred in the water column and in the surface sediments (Melendez et al., 2013a,b). Biomarkers such as isorenieratane indicative of green-brown sulfur bacteria (GSB) indicated the pivotal role that anaerobic photosynthesis played in the exceptional soft tissue preservation of this ancient crustacean. *δ*^13^C of cholestane (−30.5 ‰), short-chain C_17_/C_19_ *n*-alkanes (−34.8 ‰) and phytane (−34.0 ‰) support a source from phytoplankton consumed by the crustacean; the different values for these compounds reflect differences in biosynthetic pathways for different compound classes within phytoplankton cells (Schouten et al., 1998). Strongly ^13^C-depleted values of long chain *n*-alkanes (average-40 ‰) indicate a source from autolithified sulfate reducing bacteria (SRB) involved in concretion formation (Melendez et al., 2013b).

Another world-renowned concretion-bearing fossil site is the upper Carboniferous Mazon Creek site of Illinois, USA, hosted in the Francis Creek Shale Member of the Carbondale Formation (Johnson and Richardson, 1966; Woodland and Stenstrom, 1979; Baird et al., 1985a,b, 1986; Archer et al., 1995; Clements et al., 2018). The fossils preserved at this site include exquisitely and rarely preserved jellyfish, anemones, and shark-egg sacs, among a diverse deltaic and marine flora and fauna (Baird et al., 1985a, 1986; Clements et al., 2018; Plotnick et al., 2023). Recent geochemical studies on fossils from this site have informed the debate over the phylogenetic position of the enigmatic “Tully Monster” (McCoy et al., 2016, 2020). It has also been demonstrated that the encapsulation of coprolite fossils at this site was sufficiently rapid that intact dietary sterols were preserved (Tripp et al., 2022). An abundance of cholestane (86–99% of total steranes) alongside other cholesterol-derived compounds, including unaltered 5α-cholestan-3β-ol and coprostanol, were detected within the coprolite, whereas these compounds were comparatively low in abundance in the surrounding concretionary matrix (Tripp et al., 2022). This supports the interpretation that these compounds were derived from the original fecal material, and therefore could be treated as informative of the producer’s dietary habits. Whereas these compounds were determined to be derived from animals, supporting a primarily carnivorous diet for the coprolite producer, the *n*-alkanes were identified as of palaeoenvironmental origin. The cholestane was slightly ^13^C-depleted compared to *n*-alkanes, of palaeoenvironmental sources, and bulk OM (Tripp et al., 2022).

Moving into the Mesozoic, the Lower Jurassic Sachrang Formation of Holzmaden, Germany (often dubbed the Posidonia Shale) has produced countless exceptionally preserved fossils in concretions, including an ichthyosaur bone that preserves intact cholesterol (Plet et al., 2016, 2017). In addition, the well-preserved internal tissue of the bone included structures that resemble white- and red-blood cells (RBCs) (Plet et al., 2017). The cell-like structures interpreted as RBCs are ~20% the size of modern mammal RBCs, and their small size was explained as an evolutionary adaptation to low atmospheric oxygen levels during the Jurassic period. However, several arguments against the interpretation of these structures as ichthyosaur RBCs have been put forth: some have noted that extant reptile RBCs are considerably larger than those of mammals, and therefore much bigger than the supposed ichthyosaur RBCs (Eriksson et al., 2022; Senter, 2022); others have observed that reptilian RBCs are nucleated, unlike non-nucleated mammalian RBCs (Eriksson et al., 2022); and still others have posited that small RBCs could be inconsistent with the pelagic, deep-diving lifestyle inferred for ichthyosaurs, a lifestyle normally accompanied by an increase in RBC size (Sander, 2021; Sander and Wintrich, 2021). The cell-like structures described by Plet et al. (2017) have alternatively been linked to degraded, collagen-rich connective tissue (Senter, 2022), although their true identity remains an open question and therefore requires further research.

Several Mesozoic deposits in the Americas are known to host fossiliferous carbonate concretions in shales [e.g., the Upper Jurassic Cordillera de Domeyko Fromation in Chile (Schultze, 1989; Arratia and Schultze, 1999); the Lower Cretaceous black shales of Magdalena Valley, Colombia (Weeks, 1953, 1957)], although perhaps the most renowned is the famous Lower Cretaceous Santana Group of northeast Brazil (Mabesoone and Tinoco, 1973; Martill, 1988, 1989, 1990, 1993, 2007; Maisey, 1991; Fara et al., 2005; Varejão et al., 2021). The preservation quality of this site is sufficiently exceptional that even the hearts of fish are fossilized (Maldanis et al., 2016).

Among Cenozoic deposits with fossiliferous concretions, the Eocene London Clay Formation of the UK is perhaps one of the best known and studied (Allison, 1988b; Huggett, 1994; Huggett et al., 2000). Concretions with organismal nuclei are also known from deposits as young as Holocene in some regions, attesting to rapid encapsulation in certain modern environments. Examples of Holocene sites yielding fossiliferous concretions include North Norfolk, UK (Pye, 1984; Pye et al., 1990; Allison and Pye, 1994), Onondaga Lake, New York (Dence, 1956; Sondheimer et al., 1966; Wilcox and Effler, 1981), and Kangerlussuaq, Greenland and Ottawa, Canada (Mojarro et al., 2022). In the case of the carbonate concretions from Greenland and Canada, the capelin fish fossils conserved within were determined to have been preserved in different depositional settings from each other, providing a unique opportunity to analyze recently formed concretions for their biomarker and fatty acid compositions (Mojarro et al., 2022). Mojarro et al. (2022) demonstrated that concretions from both sites had similar organic carbon sources and environments of deposition, but that the degree and quality of organic preservation were highly divergent: one concretion exhibited exceptional soft tissue preservation, whereas the other only showed skeletal preservation. The free and bound organic fractions of the two concretions were similarly disparate: the fossil concretion with exceptionally preserved soft tissues also contained a diversity of lipids, whereas the skeletal fossil was largely overprinted by environmental lipid signals. The *δ*^13^C of *n*-alkanes and fatty acids (C_19-30_) varied between −28 ‰ to −32 ‰, which was attributed to input from C3 plants. Short-chain fatty acids, probably of a bacterial origin (C_14-18_) showed lower *δ* values (−24 ‰ to −30 ‰).

## Concretion formation mechanisms

The formation of concretions has traditionally been explained by diffusion, carbonate inter-conversion reactions, and slow three-dimensional advection of water (Berner, 1968b; Wilkinson and Dampier, 1990). Microbial processes are becoming recognized as crucial in concretion formation as well (e.g., Curtis et al., 1972; Irwin et al., 1977; Hudson, 1978; Gautier, 1982; Coleman and Raiswell, 1995; Raiswell and Fisher, 2000; Yoshida et al., 2018), and factors such as the input and decay rate of OM, carbonate saturation, porewater velocity and rate of authigenic precipitation all contribute to the exceptional preservation seen in fossil concretions (Butts and Briggs, 2011; Gaines et al., 2012; Melendez et al., 2013b; Farrell, 2014; Wilmeth et al., 2018; Grice et al., 2019; Janssen et al., 2022). Recent research has shown that microbes and their associated metabolic activities play a crucial role in the destruction, preservation and mineralization of organic tissue (s) (Allison, 1988b; Briggs, 1995a, 2003a,b; Gehling, 1999; Sagemann et al., 1999; Krause and Jachau, 2002; O'Brien et al., 2002; Briggs et al., 2005; Raff et al., 2008; McNamara et al., 2009; Janssen et al., 2022). Raff et al. (2013) and Butler et al. (2015) demonstrated that the interactions of microbes play an important role in preservation by generation of pseudomorphs which can stabilize the carcass and protect it from destruction. In another study of Eagan et al. (2017) *Bacillus* strains isolated from decaying shrimp (*Artemia sinica*) larvae and sea urchin (*Heliocidaris erythrogramma*) embryos were recorded to destroy the soft tissue. Melendez et al. (2013b) also showed evidence for the anaerobic recycling of crustacean organic matter through microbial sulfate reduction and photosynthesis (e.g., performed by the green sulfur bacteria *Chlorobi*) via biomarker analysis. In some instances, initial, rapid heterotrophic decay leads to the release of high concentrations of cations (often calcium) to the immediate environment; this may result in the stabilization and preservation of the remaining tissues, provided that the rates of the mineralization or stabilization processes remain higher than tissue decomposition (Riley, 1997; Sagemann et al., 1999; Gupta et al., 2007; Janssen et al., 2022). So, the rate of OM decay governs the rate of destruction, preservation and mineralization of organic tissues in concretion formation. The occurrence of soft tissue fossils in carbonate concretions is therefore enigmatic, as concretions are proposed to form as a result of the balanced interactions between decaying OM, microbes and the local chemical environment (Weiner and Dove, 2003; Dupraz et al., 2009; Melendez et al., 2013b).

### Role of microbes in concretion formation

Biologically induced mineralization (BIM) with organic matter (OM) is the most relevant process for fossilization and has been extensively studied by researchers over the last century (Weiner and Dove, 2003; Dupraz et al., 2009; Wilmeth et al., 2018; Dong et al., 2022). BIM is the process by which microbial metabolic activities promote mineral precipitation as a result of biochemical changes around the cell surface (Dhami et al., 2013b; Zhu and Dittrich, 2016; Murugan et al., 2021). For example, hydrolysis of urea by bacterial urease leads to an increase in pH, which supports the precipitation of calcium carbonate (Dhami et al., 2013b; Zhu and Dittrich, 2016). BIM can play a role in fossilization by interacting with decaying OM, including extracellular biopolymers or by-products of the microbial metabolic processes (Weiner and Dove, 2003; Dupraz et al., 2009; Zhu and Dittrich, 2016; Wilmeth et al., 2018). The surrounding environment of this decaying OM can act as a source of ions, influencing the chemical composition of precipitated minerals by development of appropriate conditions for free cation attachment to the nucleation sites, which are often degrading OM (Reitner, 2004; Li et al., 2015; Janssen et al., 2022).

The process of concretion formation is in essence a two-step process: (1) microbial communities adhere to the substrate, leading to the formation of a biofilm; and (2) the biofilms act as a nucleation site which, in the presence of soluble calcium ions under a high pH environment, results in biomineralization of calcium carbonate (Weiner and Dove, 2003; Dupraz et al., 2009; Melendez et al., 2013b).

During BIM, biofilm formation occurs with the adhesion of microbial communities on surfaces, which combine to form clusters or microcolonies. Microcolonies progress to a threshold density and form a biofilm, which then induces the expression of biosynthesis genes that control the synthesis of extracellular polymeric substances (EPS); these EPSs then become encapsulated within the biofilm (Costerton et al., 1995; Percival et al., 2011; Janssen et al., 2022). Biomineralization usually occurs on biofilms with heterogeneous structures because of cell aggregation (Li et al., 2015). With these differential structures, the microenvironment both within and external to the biofilm is modified to facilitate mineralization (Iniesto et al., 2015, 2016, 2018). For example, the biofilm formation of *Pseudomonas aeruginosa* accumulates minerals on the inside and outside of the biofilm by trapping fine abiotic calcite particles as well as granules of calcite through biomineralization (Bai et al., 2017). Biofilms are also known to exhibit diverse and highly structured microenvironments resulting from a combination of microbial metabolism and transport limitations (Stewart and Franklin, 2008). Li et al. (2015) demonstrated different patterns of biomineralization *in situ* with precipitation starting at the base of the biofilm and building upwards which impacts mineralization. *Ex situ* studies suggest that the extracellular polymeric substances (EPS) produced by biofilms influence precipitation and regulate patterns of mineralization (Ercole et al., 2007). Biofilm morphology also regulates the internal and external solute transport mechanisms, which determines the physiology of the biofilm-resident cells. Previous studies have demonstrated that microbial biofilms surrounding dead and decaying biomass also act as a template for carbonate precipitation and concretion formation (Iniesto et al., 2015, 2016).

Mineralization requires supersaturation of specific ions in the surrounding environment which can be governed by microbial activity in and around the cadaver immediately upon death. Depending upon the availability of organic substrates and cations, different mineralization reactions can occur, including silicification, phosphatization, pyritization or carbonate precipitation (Briggs et al., 1996; Grimes et al., 2001; Dornbos, 2011; Gaines et al., 2012; Farrell, 2014; Liu D. et al., 2020; Janssen et al., 2022). Among these, carbonates are the most commonly precipitated biominerals (Thompson et al., 1997; Grotzinger and Knoll, 1999; Zhu and Dittrich, 2016).

**Figure 4:**
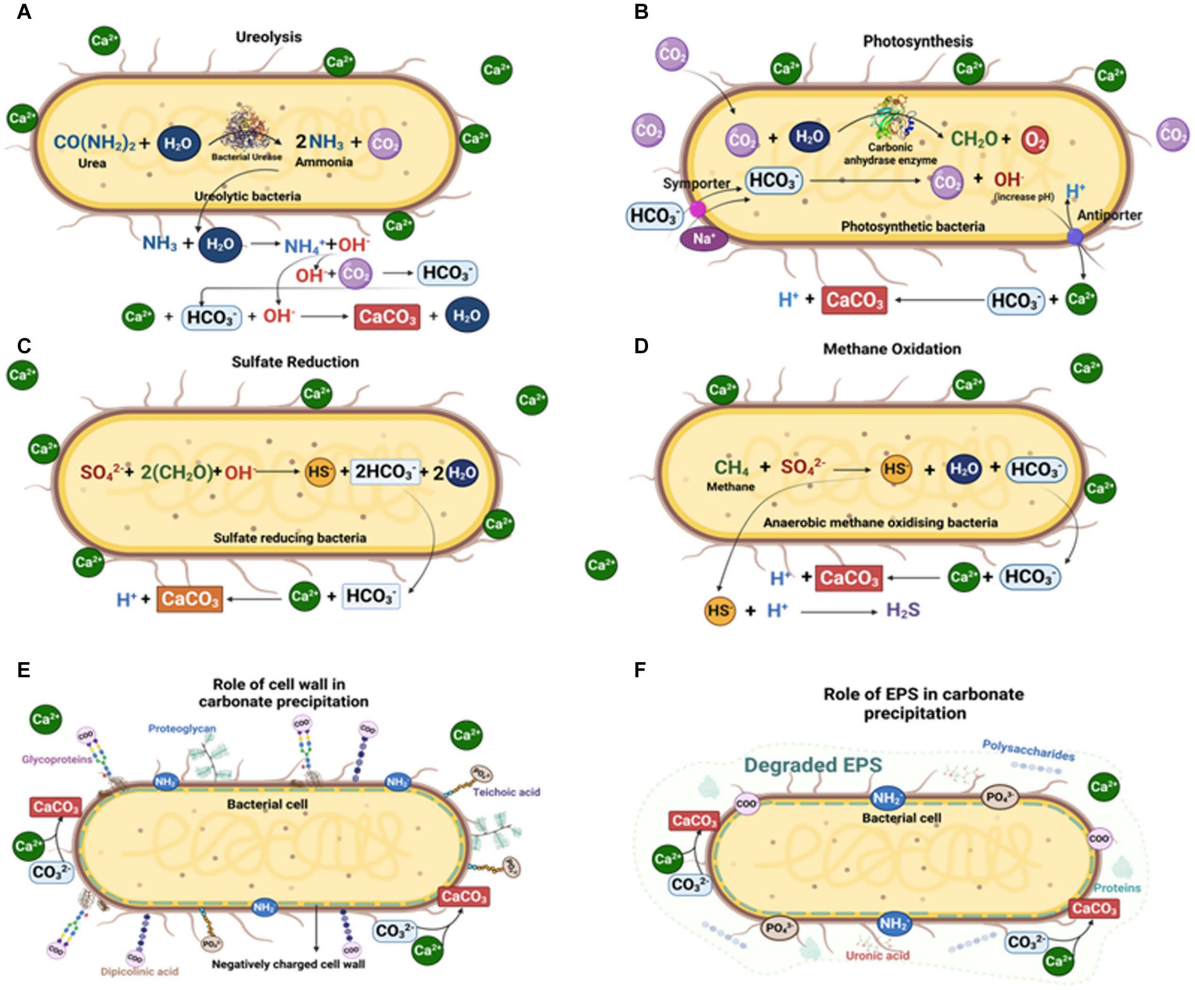
Microbially mediated carbonate concretion formation by different microbial pathways: **(A)** Ureolytic pathway; **(B)** Photosynthetic pathway; **(C)** Sulfate reduction pathway; **(D)** Methane oxidation pathway; **(E)** Carbonate precipitation on microbial cell walls; and **(F)** Carbonate precipitation via microbial EPS (Created with BioRender.com).

Microbial function and metabolic activity in biomineral precipitation (especially carbonates) can be grouped into three categories (Figure 4):

- Precipitation of microbial metabolic pathway by-products. Metabolic pathways like photosynthesis, sulfate reduction, denitrification, anaerobic sulfide oxidation, ureolysis and methanogenesis might circumstantially lead to mineralization (Konhauser et al., 2005; Zhu and Dittrich, 2016; Zeng and Tice, 2018). The significance of SRB, iron reducing bacteria and methanogens has been widely recorded in calcite and siderite concretions (Coleman and Raiswell, 1981; Curtis et al., 1986; Coleman, 1993; Melendez et al., 2013a,b; Zeng and Tice, 2014; Cotroneo et al., 2016; Gaines and Vorhies, 2016; Plet et al., 2016; Lengger et al., 2017).
- Interaction of the cell wall with the environment. Carbonate nucleation takes place either through the cell wall via ion exchange, or on the cell wall because of the presence of negatively charged functional groups like carboxyl, phosphate and amine, which can bind to divalent cations (e.g., Ca^2+^, Mg^2+^) when available (Warren et al., 2001; Dittrich and Sibler, 2005; Ercole et al., 2012). Following the increase in concentration of these metal ions in the surrounding microenvironment, and with sufficient availability of bicarbonates/carbonates, an oversaturation of carbonates is achieved, such as in calcite precipitation on cell walls of picocyanobacteria (Warren et al., 2001).
- Entrapment by extracellular polymeric substances (EPS). EPS mainly comprise polysaccharides and proteins along with nucleic acids, lipids, uronic acids (Nichols and Mancuso Nichols, 2008; Ercole et al., 2012; Bains et al., 2015). As EPS contain various acidic residues and sugars, they can trap large divalent cations (e.g., Ca^2+^ and Mg^2+^) and remove free cations from solution. Once EPS degrade, the captured cations remain, and so the local concentration of cations increases and promotes calcium carbonate precipitation.

In the molecular fossil record, metabolic pathways influencing soft tissue preservation have been recorded via biomarker analysis. For example, (Melendez et al., 2013b) reported anaerobic recycling of invertebrate OM via microbial sulfate reduction and photosynthesis. Taphonomic experiments demonstrated the decay of arthropod brains in several Cambrian fossil sites, confirming the role of bacterial activity in mineralization (Parry et al., 2018; Purnell et al., 2018). Further evidence has been recorded in the taphonomic studies of Butler et al. (2015) and Bickel and Tang (2010) on bilaterians. It was found that, in order to counterbalance the rapid gut biota-driven internal decay of bilaterian tissues, gut biota rapidly escape the gut after death and form internal biofilms; these eventually provide a template for, and induce, mineralization, which facilitates concretion formation and the preservation of soft tissue (Bickel and Tang, 2010). The precipitation of siderite concretions has also been attributed to a complex array of sulfate and iron reducing bacteria (Farrell, 2014; McCoy, 2014; Janssen et al., 2022). Bone marrow and skin structures in frogs preserved on microbial mats for 3 years have been recorded in earlier studies (Iniesto et al., 2013, 2016, 2017). Previous studies have also demonstrated that microbes promote concretion formation via mineral precipitation on decaying carcass or within the sediment around decaying tissues (Pye et al., 1990; Briggs et al., 1993; McCoy et al., 2015b). All these investigations provide valuable evidence highlighting the role of microbes in several concretions and fossilization processes, but many other governing factors also play a crucial role.

### Physicochemical factors influencing microbially mediated concretions

Numerous physicochemical parameters influence the formation and composition of microbially mediated concretions, a fact attested to by the variable size (both absolute and relative to the concretion nucleus) and geochemistry of concretions (and the fossils they host) in the fossil record. Several of these physicochemical factors are explored below.

#### Anoxic conditions

Successful fossilization typically correlates with anoxic conditions attributed to rapid burial, which concomitantly prevents scavenging of OM and inhibits aerobic decay processes (Allison, 1988a). Instead, OM decomposition proceeds via anaerobic metabolic pathways (Briggs, 2003b; Janssen et al., 2022). Moreover, under anaerobic conditions, denitrification (e.g., in *Pseudomonas* sp.), sulfate reduction to H_2_S, and anaerobic methane oxidation (coupled with sulfate reduction) are known to increase the alkalinity and change the chemistry around cells leading to carbonate precipitation (van Rijn et al., 2006; Zhu and Dittrich, 2016). In carbonate concretions, early diagenetic decay-induced mineralization is considered responsible for the co-occurrence of concretion formation with soft tissue fossils at their center. Some studies have suggested that anaerobically driven microbial processes, such as sulfate reduction and production of H_2_S, have been critical in facilitation of exceptional soft tissue fossil preservation (e.g., Melendez et al., 2013a,b; Schwark, 2013; Plet et al., 2016; Lengger et al., 2017).

#### pH

The chemistry of pore waters is a significant control in mineral precipitation, and consequently in carbonate concretion formation. Berner (1968a) reported that decaying tissue can increase pH as well as promote precipitation of calcium and magnesium from dissolved ions in solution. This rise in pH is related to the degradative release of bases such as ammonia (Berner, 1968a). However, further work by Berner (1969) showed that calcium carbonate precipitated only when the pH rise coincided with extensive bacterial sulfate reduction (BSR). pH also plays a vital role in the carbonate–phosphate switch that controls carbonate precipitation. Under low-pH conditions (<pH 6.38), and in the presence of free calcium ions and phosphate ions, calcium phosphate precipitates; by contrast, at higher pH (> pH 6.38), and in the presence of free calcium and carbonate ions, calcium carbonate precipitates (Briggs and Kear, 1993c; Briggs and Wilby, 1996; Janssen et al., 2022).

#### Other physico-chemical variables

Other factors including dissolved oxygen (DO), conductivity and temperature have been demonstrated to be contributing variables in concretion formation (e.g., Briggs, 1995b; Iniesto et al., 2017; Muramiya et al., 2022). Glendonite, a calcite pseudomorph after ikaite (CaCO_3_.6H_2_O), forms during early diagenesis in marine sediments and under relatively low temperatures ranging from-2°C to 10°C. This biomineral has been widely used as a low-temperature indicator to reconstruct paleoclimate (De Lurio and Frakes, 1999; Muramiya et al., 2022; Rogov et al., 2023). According to a study by Iniesto et al. (2017), DO exhibited a control similar to pH during laboratory based taphonomic experiments on frogs. The DO and pH were stabilized after seven weeks of incubation of a frog, and the drop in DO and pH implies the period of mineralization. This clearly demonstrated that DO and pH has a vital, but as yet unpredictable role in mineralization.

### Microbiological controls on carbonate precipitation and concretion formation

Carbonate mineral precipitation is controlled by a number of complex, interrelated chemical processes. This includes alkalinity changes induced by microbiological processes, which can be dictated by the local environment and pore water chemistry (e.g., Berner, 1968b), or influenced by bicarbonate ions sourced from the surrounding pore waters or from decaying OM (Curtis et al., 1986). A source of cations within the substrate and/or porewaters is also required for concretion precipitation, most commonly calcium or iron forming calcite and siderite concretions, respectively (Curtis et al., 1986; Marshall and Pirrie, 2013; McCoy, 2014). Dolomitic concretions can also form in magnesium-rich environments (e.g., Grice et al., 2019; Scheller et al., 2021). Commonly initiated by decay of a central OM-rich nucleus, carbonate cement precipitates within unconsolidated sediment (Raiswell, 1971; Raiswell and Fisher, 2000; McCoy, 2014; Lengger et al., 2017). Carbonate concretions can form either via pervasive growth where nucleation is simultaneous across the concretion and cementation infills pore space later; concentric mineral growth outwards from a central nucleus; or by a combination of both processes (Raiswell and Fisher, 2000). The role of microbiological OM oxidation in concretion initiation and growth has been demonstrated by stable carbon isotope analyses (e.g., Curtis et al., 1972; Hudson, 1978; Coleman and Raiswell, 1981; Gautier, 1982; Raiswell and Fisher, 2000; Cotroneo et al., 2016), as well as biomarker studies (e.g., Melendez et al., 2013a,b; Plet et al., 2016; Lengger et al., 2017). Previous studies have also proven the role of bacterial EPS in mineral precipitation, for example absorbance of iron from iron-rich chlorides and oxides onto the surface of EPS during authigenic precipitation (Spicer, 1991; Dunn et al., 1997).

#### Calcium carbonate concretions

Calcium carbonate (calcite; CaCO_3_) precipitation is a consequence of BSR, wherein sulfate reducing bacteria oxidize OM to CO_2_ to obtain energy and reduce sulfate to H_2_S (Coleman and Raiswell, 1981; Coleman, 1993; Coleman and Raiswell, 1995; Kiriakoulakis et al., 2000; Melendez et al., 2013b; Plet et al., 2016). BSR is chemically represented by Equations 1, 2 (Raiswell, 1976). Subsequently, reduced sulfide reacts with dissolved iron to form pyrite via solid iron monosulfide (Equations 3, 4; e.g., Berner, 1985), while carbonate reacts to form calcite (e.g., Berner, 1968a; Pye et al., 1990).


(1) $$\text{BSR:}\;2\;{CH}_{2}O+{{\mathrm{SO}}_{4}}^{2-}\to 2\;{{HCO}_{3}}^{-}+H_{2}S$$

(2) $$2\;{CH}_{2}O+{{\mathrm{SO}}_{4}}^{2-}\to {{HCO}_{3}}^{-}+{CO}_{2}+{HS}^{-}+H_{2}O$$

(3) $$\text{Pyrite formation:}\;{Fe}^{2+}+H_{2}S\to FeS+2\;H^{+}$$

(4) $$FeS+H_{2}S\to {FeS}_{2}+H_{2}$$

**Figure 5:**
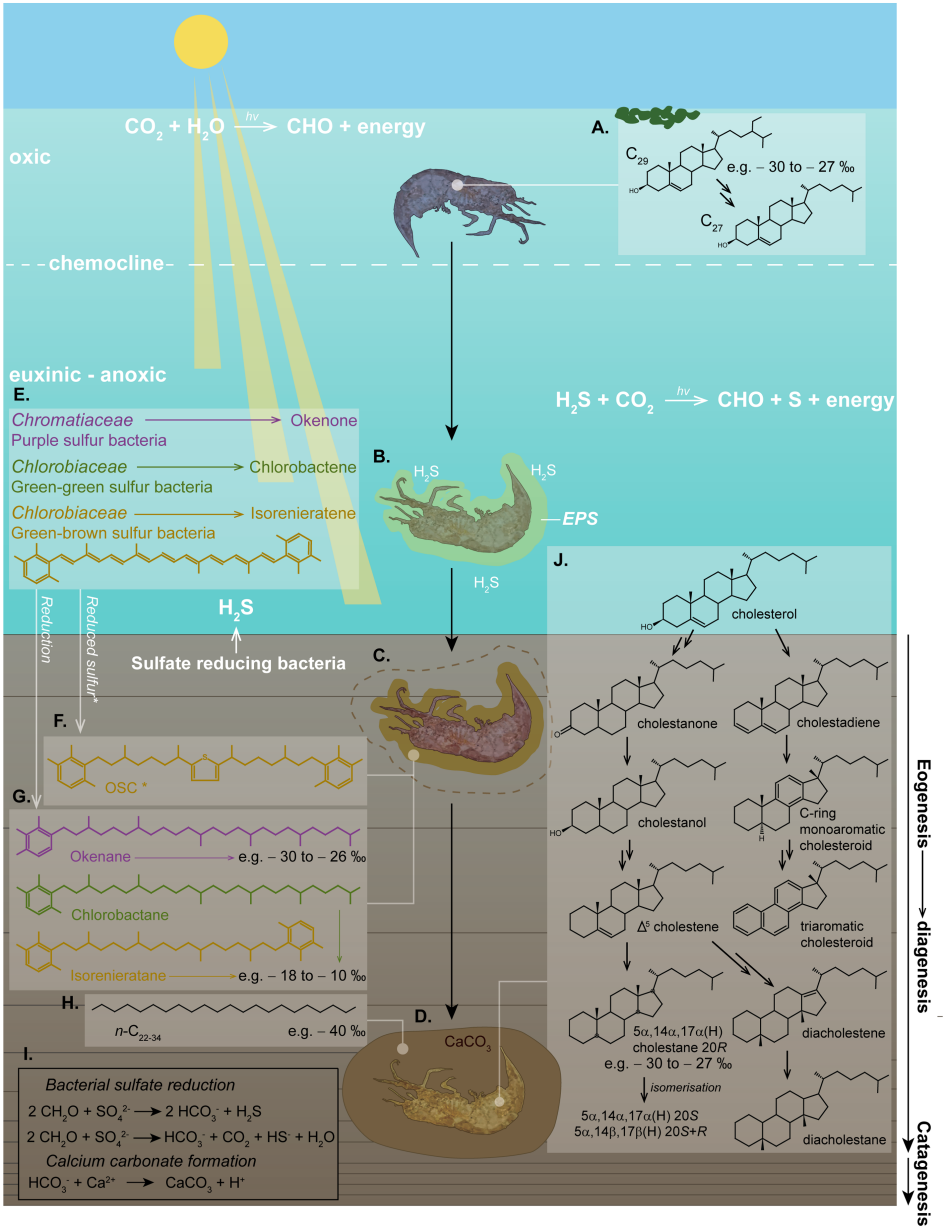
Schematic of photic zone euxinia conditions, calcium carbonate concretion formation and *in-situ* fossilization, demonstrating the complex eogenetic (water column) and diagenetic (sediment/water interface) processes which can be interpreted from molecular biomarkers. **(A)** An OM nucleus (e.g., a shrimp) in the water column can produce cholesterol by dealkylating sterols (e.g., C_29_ stigmasterol) from an algal diet. The δ^13^C value of this cholesterol is representative of the average δ^13^C value of the sterols from the dietary sterols in algae. **(B)** Toxic H_2_S might have caused the death of the organism. Bacterially derived EPS build an envelope around the decaying carcass. **(C)** EPS promote calcium carbonate precipitation around the nucleus, halting further OM degradation early in diagenesis. **(D)** The carcass becomes fully encapsulated in the calcium carbonate matrix, promoting rapid (days–weeks–months) preservation of soft tissue and biomarkers/biomolecules. **(E)** Where light reaches the anoxic zone of a stagnant water column and H_2_S produced by bacterial sulfate reducers reaches the sunlit zone, PZE conditions develop, where anaerobic phototrophs can flourish. These include Chromatiaceae (purple pigmented sulfur bacteria) and Chlorobiaceae (green-green and green-brown pigmented sulfur bacteria) in a distinct zonation, which synthesize specific carotenoid pigments, including (but not limited to) okenone, chlorobactene, and isorenieratene, respectively. **(F)** These carotenoids can be incorporated into organosulfur compounds (OSCs) intermolecularly or intramolecularly bound in the soft tissue, or; **(G)** they can be reduced to stable hydrocarbons. Both of these processes provide specific biomarkers which can indicate the role of PZE and SRB in calcium carbonate formation and fossil preservation. **(H)** *n*-alkanes with depleted δ^13^C values (e.g., −40 to-36 ‰) can be indicative of SRB. **(I)** Chemical reactions of bacterial sulfate reduction forming H_2_S, and calcium carbonate precipitation. **(J)** Diagenetic breakdown of cholesterol into stable steroids via a range of intermediates, illustrating diagenesis as described by Mackenzie et al. (1982). Structures shown represent the full suite of diagenetic breakdown products of cholesterol as identified in a calcium carbonate concretion containing a crustacean by Melendez et al. (2013a). The high percentage abundance of cholesteroid biomarkers was used to identify the fossilized organism as a crustacean. This study was demonstrative of the wealth of information which can be extracted from organisms which can be preserved under favorable conditions, such as within carbonate concretions.

Initial stages of calcite concretion formation typically occur within the earliest stages of diagenesis. Anoxic marine settings have abundant dissolved sulfate and therefore favor calcite precipitation (Berner, 1985). H_2_S at the photic zone of an anoxic water column promotes photic zone euxinia (PZE), which has been demonstrated to facilitate exceptional OM preservation (see Figure 5) (e.g., Summons and Powell, 1987; Schwark and Püttmann, 1990; Grice et al., 1996, 1997; Schaeffer et al., 1997; Figure 4). Notably, calcite concretions preserving soft tissue and biomolecules have been associated with PZE (e.g., Melendez et al., 2013a,b; Plet et al., 2016; Lengger et al., 2017).

Calcite concretions can display varying morphological and mineralogical properties with different crystalline phases. These include, but are not limited to, calcite, aragonite, vaterite, dolomite, CaCO_3_ monohydrate, CaCO_3_ hexahydrate and amorphous CaCO_3_ (Rodriguez-Navarro et al., 2012; Murugan et al., 2021). The shape and size of CaCO_3_ crystal changes during the precipitation process under different physico-chemical conditions and in association with different microbial communities (Wilby et al., 1996; O'Brien et al., 2002; Raff et al., 2008; Dhami et al., 2012; Raff and Raff, 2014; Plet et al., 2016; Zhu and Dittrich, 2016; Iniesto et al., 2018; Grice et al., 2019; Dubey et al., 2022; Murugan et al., 2022). The major factors responsible for microbially induced concretion precipitation include the concentration of calcium, concentration of DIC, pH, and the availability of nucleation sites (Hammes and Verstraete, 2002; Dhami et al., 2013b; Murugan et al., 2021).

#### Iron carbonate concretions

In contrast to calcite-forming environments, siderite precipitation is associated with sulfate-limited environments (Coleman, 1993), where dissolved sulfate is rapidly utilised forming pyrite (Equations 3, 4), and bacterial metabolic processes mainly occur via microbial iron reduction (Curtis et al., 1986) and methanogenesis (Maynard, 1982; Curtis et al., 1986; Coleman, 1993; Janssen et al., 2022), as per Equations 5, 6, respectively. This is observed in freshwater systems, such as lakes and swamps.


(5) $$\begin{array}{l} \mathrm{Iron reduction}:4\;\mathrm{FeOOH}+{CH}_{2}O+7\;H^{+}\to 4\;{Fe}^{2+}+{{HCO}_{3}}^{-}\\ \;+6\;H_{2}O \end{array}$$

(6) $$\mathrm{Methanogenesis}:2\;{CH}_{2}O+H_{2}O\to {{HCO}_{3}}^{-}+{CH}_{4}+H^{+}$$

Pyrite formed as a product of early BSR is often identified in relation to siderite concretions; for example, in siderite concretions from Mazon Creek, pyrite can be observed either localized with fossils or in halos around them (e.g., Cotroneo et al., 2016). In the case of Mazon Creek siderite concretions, δ^34^S isotope data indicated initial bacterial sulfate reduction rapidly gave way to pervasive siderite growth via methanogenesis (Cotroneo et al., 2016).

**Figure 6:**
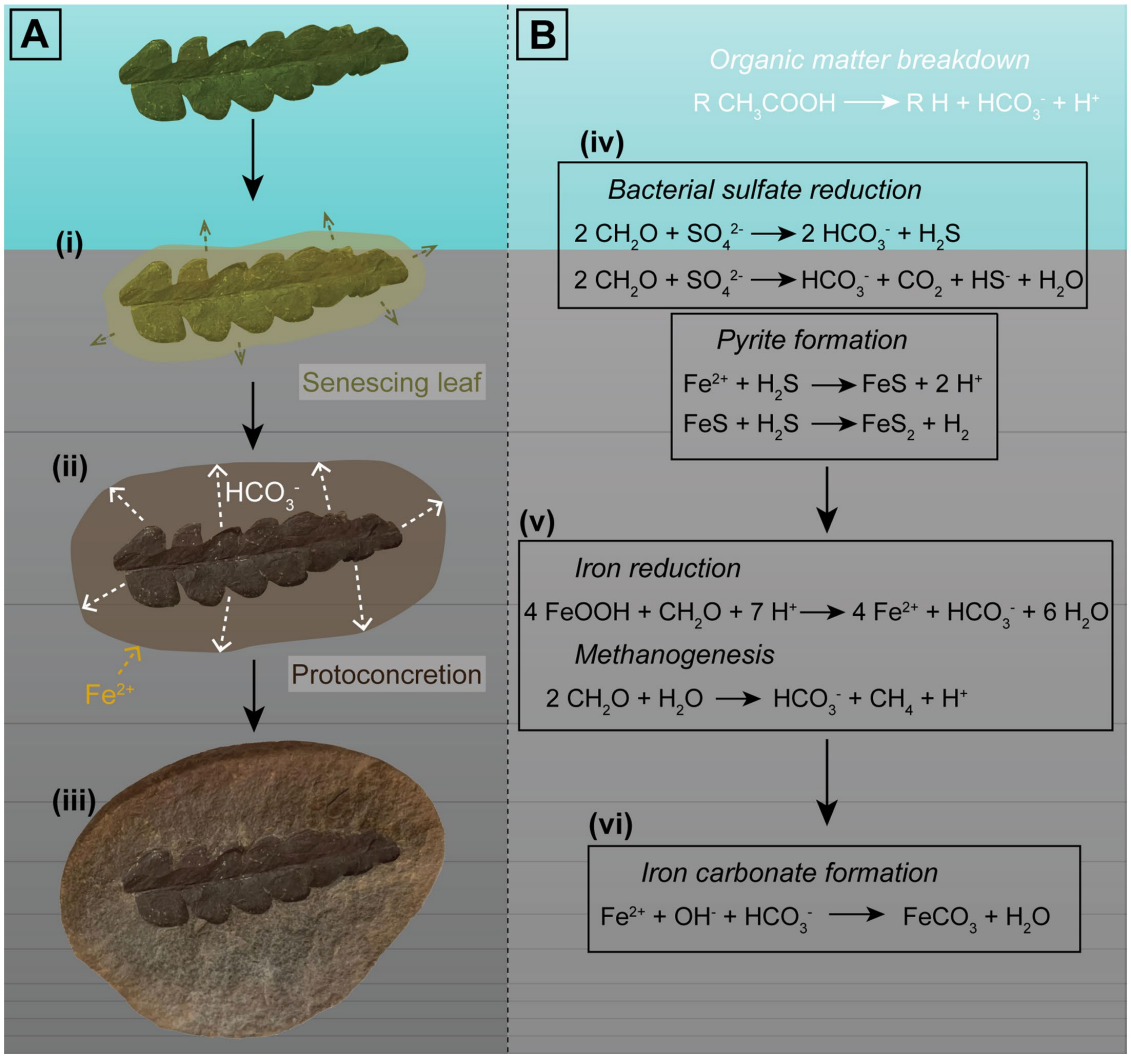
Visual representation of the factors involved in formation of iron carbonate concretions in freshwater influenced environments. Sample used as an example is an iron carbonate concretion from the Mazon Creek Lagerstätte containing an *Odontopteris aequalis* seed fern. **(A)** Proposed phases of concretion growth promoted by decay of an OM source: (i) An organic nucleus, such as a leaf, is deposited near the sediment–water interface, and decay results in OM breakdown; (ii) Oxidized OM forms bicarbonate ions, which seep outwards (e.g., Yoshida et al., 2015, 2018), which could then react with Fe^2+^ in surrounding pore-waters. Siderite precipitation forms a ‘proto-concretion’, encapsulating the specimen and the OM; and (iii) Siderite cementation results in formation of a nodule containing a soft tissue fossil. **(B)** Equations representing the chemical reactions involved in OM oxidation and carbonate formation: (iv) In settings such as freshwater environments, where sulfate is limited, BSR may or may not occur. When it does, it is dependent on sulfate abundances in the pore-water and proceeds only until sulfate is consumed. The reduced sulfate will react with iron and form pyrite via iron monosulfide (e.g., Berner, 1985); (v) Once bacterial sulfate reduction ceases, OM oxidation occurs via iron reduction and methanogenesis; and (vi) This (provided conditions such as pH are suitable) promotes iron carbonate precipitation. Carbonate concretion growth is proposed to proceed until the OM is exhausted (e.g., Baird et al., 1986; Yoshida et al., 2015, 2018).

Lipids released from decaying organisms might also play a potential role in promoting microbial processes (Kamran et al., 2020). Pure cultures of the iron-reducing bacteria *Geobacter* and *Shewanella* have precipitated siderite under laboratory conditions (Lin et al., 2020; Janssen et al., 2022). The reduction of the labile iron can raise the pH above optimal conditions (precipitation of calcite and aragonite is favored over pH ~7.2); however, when coupled with low levels of sulfate reduction, pH variation is limited within the range of siderite precipitation (Lin et al., 2020; Janssen et al., 2022). Here, iron (III) is used as an electron source for the oxidation of OM to CO_2_ instead of sulfate, by sulfate reducers such as *Desulfovibrio* (Kamran et al., 2020). A detailed model of iron carbonate concretion formation is shown in Figure 6.

#### Phosphatic concretions

Marine phosphatic concretions are formed under anoxic conditions near the sediment–water interface, during early diagenesis. Phosphate is sourced from the sediment pore waters with bacteria playing a crucial role in phosphate fixing, with a possible role for bacterial mats in concretion mineralization (Baturin, 1971; Bentor, 1980; Reimers et al., 1990). Large sulfur bacteria like *Beggiatoa* or *Thiomargarita* can store phosphate in the form of polyphosphate under oxic conditions (Brock and Schulz-Vogt, 2011). However, these bacteria produce sulfide, and if oxygen supply is insufficient for complete oxidation of the sulfides, the capacity for polyphosphate storage decreases. Increasing sulfide concentrations and anoxia leads to the decomposition of polyphosphate and iron hydroxides, causing the bacteria to release inorganic orthophosphate (Pi) into surrounding pore water (Brock and Schulz-Vogt, 2011). Pi is the precursor for the precipitation of phosphorite minerals and phosphatic replacement of soft tissues (Janssen et al., 2022). Once precipitation begins, the process is estimated to phosphatize soft tissues rapidly, in timescales of days to weeks (Föllmi, 1996).

Most of these phosphatized concretions are triggered by the decomposition of OM by subsurface microbial communities involved in bacterial sulfate reduction and anaerobic methane oxidation, EPS formation, photosynthesis, ureolysis, iron reduction (Equations 3, 4, 7; Plet et al., 2016; Zhu and Dittrich, 2016; Kamran et al., 2020).


(7) $$M^{2+}+{{2HCO}_{3}}^{-}\to {MO}_{3}+H_{2}O+{CO}_{2}$$

The conditions of phosphatization involve phosphate ions (from the decay of animal remains or an allogenic source), Ca^2+^ ions from seawater and low pH from the production of volatile fatty acids and CO_2_ (Zoss et al., 2019). Phosphatized concretions are formed from carbonate fluorapatite (Ca_5_(PO_4_,CO_3_)_3_F) and occur most in deposits of Jurassic–Cretaceous age, when phosphogenic-favoring environments were particularly prevalent (Martill, 2007; Dornbos, 2011). Phosphatic concretions have been identified in deposits ranging in age from upper Mesoproterozoic [e.g., Diabaig Formation of the Torridon Group, Scotland, UK: Battison and Brasier (2012)] to Miocene, e.g., Riversleigh World Heritage Area, Queensland, Australia (Arena, 2008; Matzke-Karasz et al., 2013) and Funakawa Formation, Japan (Ogihara, 1999). Numerous modern phosphatic-rich deposits are found in organic-rich, offshore environments, e.g., Baja California (Schuffert et al., 1994; Ogihara, 1999), the Namibian coast (Zoss et al., 2019) and the Chilean-Peruvian coastline (Turnbull et al., 1996; Schulz and Schulz, 2005). A famous example from the fossil record is the Lower Cretaceous Santana Group Lagerstätte of Brazil, which preserves an array of fossils including abundant invertebrates, teleost fish, crocodyliforms, pterosaurs, and rare dinosaurs (Smith, 2000; Martill, 2007; Martill and Brito, 2017). Skin, gills, muscles, and collagen are the most frequently phosphatized soft tissues (Vincent et al., 2017; Parry et al., 2018). Phosphatization is also able to preserve cellular and subcellular structures (Bengtson and Budd, 2004; Bengtson et al., 2017; Sun et al., 2020). In bone tissues, the outer compact bone layers are more prone to replacement with fluorapatite, but the inner spongy bone is more prone to be replaced with calcite; however, the timing of phosphatization of bone tissues is still not fully understood (Zoss et al., 2019). The creation of phosphatized tissues and phosphatic concretions in microbial mats is also largely unknown, however there is extensive research on the role of organic-rich substrate in the precipitation of calcium phosphate (Onuma et al., 2000; Heinemann et al., 2011; Janssen et al., 2022). One location reported to contain microbial mat-preserved fossils is the Crato Formation *Konservat-Lagerstätte* of the Santana Group, Araripe Basin, Brazil (Varejão et al., 2019).

## Analytical methods for concretion characterization

**Figure 7:**
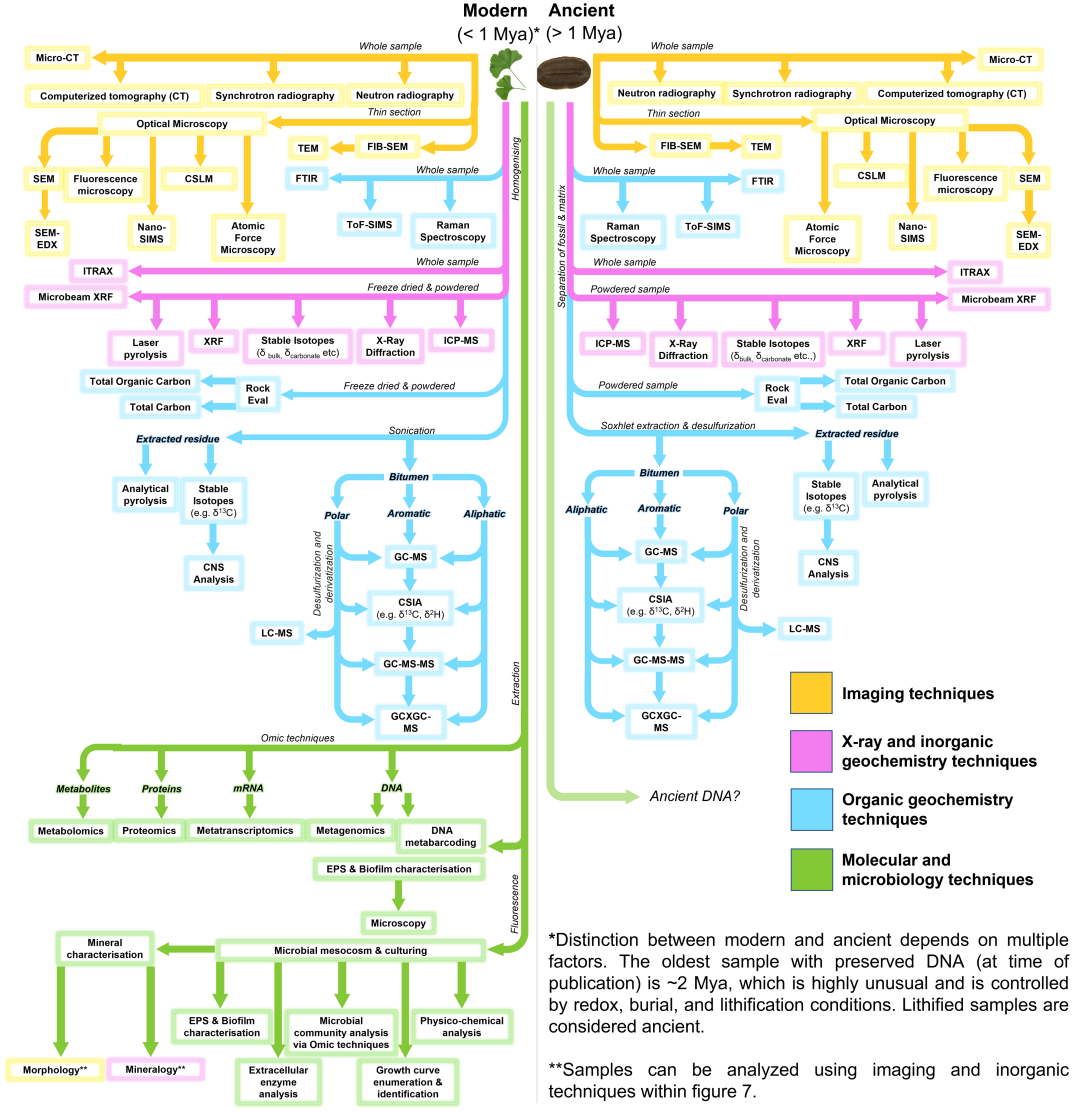
Flow diagram for analytical methods applicable to microbial fossil concretions, modern and ancient.

**Figure 8:**
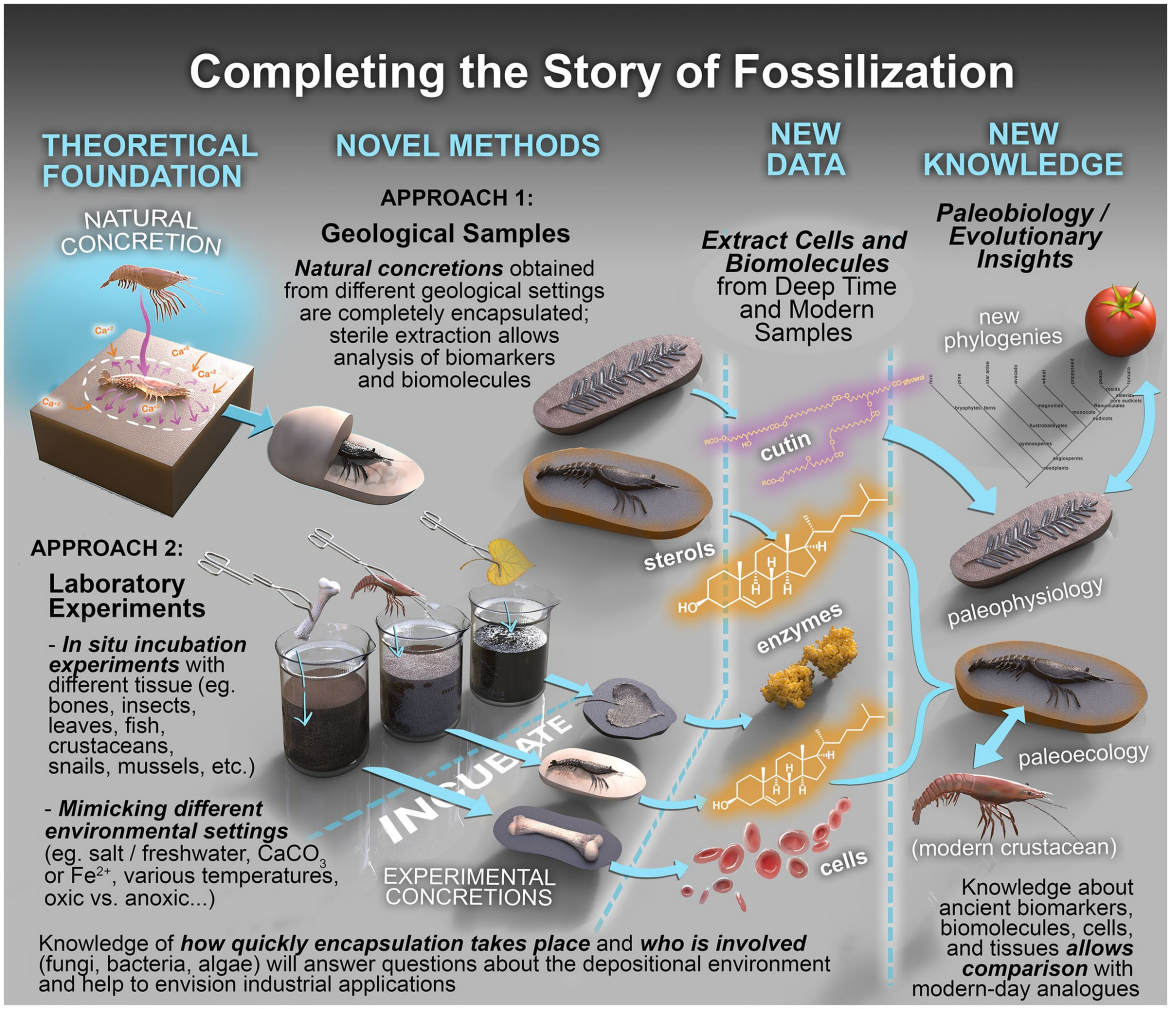
Completing the story of fossilization. Conceptual framework to establish fossilization processes and interrogate their biochemical record. The framework combines the complementary analysis of real samples (*Approach 1*) with those produced under carefully controlled laboratory conditions (*Approach 2*).

A range of sophisticated and multidisciplinary analytical approaches can be utilized in the robust interrogation of fossiliferous concretions and the fossils they contain (Figure 7; Table 2). These methods include: using powerful imaging tools to resolve the physical form and morphologies of concretions and fossils, sometimes at sub-microscopic levels; organic characterization of the nature and molecular speciation of the preserved organisms and the paleoenvironment in which they were formed; inorganic geochemical analysis for depositional information and evaluation of the redox conditions aiding preservation; and the use of microbiological and molecular techniques to study the structure and function of the microbes active in EPS and concretion formation (Figure 8: Approach 1).

**Table 2:** Brief summary of the various analytical techniques applicable to concretion analysis, as discussed in this review.

Technique	Description	Advantages	Disadvantages
Morphological analyses			
Scanning electron microscopy (SEM)	A focused beam of electrons is used to image the sample	High resolution (<20 nm)	Low electron penetration depth
SEM/EDS	SEM attached to EDS system, for spatial elemental analysis	Quantitative analysis of elements & mapping distribution	Low electron penetration depth
Focused ion beam (FIB)-SEM	Uses a focused beam of ions to form an image	Quantitative analysis of elements & mapping distribution	Destructive analysis
Optical (light) Microscopy	Visible light analysis of structures in fixed & living samples	Rapid results	Limited resolution, & not suitable for all samples
Confocal Scanning Laser Microscopy (CSLM)	Optical imaging technique using laser to increase contrast & resolution	Non-intrusive & non-destructive, minimal sample preparation	Highly limited depth of field, & slow process
Fluorescence Microscopy	Emission of luminescence to identify structures in fixed & living samples	Organic maceral identification	Limited resolution, & not suitable for all samples
Atomic Force Microscopy (AFM)	Optical imaging technique, does not use lenses or beam irradiation	Very- high resolution (~nm), no need to stain or place sample in vacuum	Small single scan image size, & slow process
X-Ray Fluorescence (XRF)	Characterization of the elemental composition of bulk sediment	Can analyze wide range of elements	Cannot identify different phases
X-Ray Diffraction (XRD)	Characterization of mineralogical composition	High precision & non-destructive	Requires a homogenous sample
Computed tomography (CT)	Imaging & quantification of 3D structures using X-ray microscopy	Non-destructive, can scan large samples	Lower resolution than other tomography-based methods
Micro-CT (μCT)	Imaging & quantification of 3D structures using X-ray microscopy	High resolution, non-destructive	Limited sample size (chamber), & long scanning times
Synchrotron propagation phase contrast micro-CT (PP-SRμCT)	3D imaging technique	Non-destructive, rapid, high-spatial resolution	Complex process requiring multiple stages, samples require mounting
Neutron scattering	Imaging of 3D structures to see hydrogenous materials	Greater sample penetration than X-rays	Potential radioactive decay induced in samples
Organic geochemical (and related) analyses			
Gas chromatography–mass spectrometry (GC–MS)	Identification & quantification of compounds in organic mixtures	Widely available, mature technology for light, non-polar compounds	Destructive analysis, often intricate sample preparations, analysis & data interpretation
Tandem mass spectroscopy (GC MS–MS)/Multiple Reaction Monitoring (MRM)	Targeted GC–MS analysis of known compounds	Increased sensitivity & selectivity of targeted compounds (e.g., biomarkers)	Requires advanced MS instrumentation & prior GC–MS analysis to identify targets
Multidimensional GC–MS (GC x GC)	GC separation using two different column types	Greatly improved separation of compounds, including those that coelute on one-dimensional GC–MS	As for GC–MS and GC MS–MS, & high data processing times
Time of Flight-Secondary Ion Mass Spectrometry (ToF-SIMS)	Elemental, chemical state, & molecular information from solid surfaces	High mass range, & high spatial resolution over wide cross-section of sample surface	Superimposed MS of complex organic mixture; not capable of resolving isomers
Liquid chromatography-mass spectrometry (LC–MS)	Separation & analysis of large, polar compounds	Mature technology, allows analysis of compounds not amenable to GC–MS	Destructive analysis, long preparation time needed for extraction of sample
Inductively Coupled Plasma Mass Spectrometry (ICP-MS)	Trace metal analysis	Can be coupled with laser probes to create high spatial resolution elemental maps	Destructive analysis, & wider field of ion energy resulting in noisy signals
Raman spectroscopy	Information about chemical structure, phase crystallinity & molecular interactions	Non-intrusive & non-destructive	Cannot analyze samples with high topography
Bulk isotope analysis	Distinguish OM source inputs, cycling of elements	Minimal sample preparation, rapid analysis	Gives an averaged value of complex organic samples, i.e., cannot distinguish multiple sources
Compound-specific isotope analysis (CSIA)	Stable isotope mass spectrometry coupled with gas or liquid chromatography	Stable isotope values of individual compounds in complex mixture	Requires prior GC–MS identification & rigorous sample preparation for typically required baseline peak separations, & incomplete separation of a compound in the mixture can lead to isotopic fractionation
Fourier-transform infrared spectroscopy (FT-IR)	Identification of organic, polymeric, & some inorganic materials via infra-red light	Highly sensitive & rapid analysis of organic bonding & functional groups	Qualitative analysis, variable chemical species response
Atomic absorption spectroscopy (AAS)	Detects elements using the absorption wavelengths of light	High accuracy	Can only measure one element at a time, destructive analysis

### Morphological, molecular, and microbiological characterization of concretions

An understanding of the metabolic activities that took place at the time of fossilization can be obtained through various analytical techniques, using either direct (microbial morphotypes, mineralized EPS, etc.) or indirect (microlaminations with OM or micropeloids) microscopic and elemental evidence (Iniesto et al., 2021; Dias and Carvalho, 2022). This understanding can be furthered via investigation of the influence of various metabolic pathways on decay and mineralization through taphonomic experiments in controlled environment (Pye et al., 1990; Briggs and Kear, 1993c; Butler et al., 2015; Wilson et al., 2016; Gäb et al., 2020; Janssen et al., 2022). In the previous taphonomic experiments, Pye et al. (1990) demonstrated at the Norfolk Marsh field setting that concretions form actively in reduced sediments in which sulphate-reducing bacteria are active. The source of carbonate in their study was found out to be partly driven from marine sources and partly from microbial degradation of organic matter. In another study of Briggs and Kear (1993c) on decay of modern shrimps, it was seen that partial mineralization occurred in amorphous calcium phosphate leading to preservation of cellular details of the muscle tissue under anaerobic conditions wherein microbial biofilms played an important role in the inhibition of tissue decomposition. The anaerobic conditions inhibit scavengers and degrading aerobic bacteria and promote various anaerobic metabolic pathways relevant for biologically induced mineralization (Wilson et al., 2016). Gäb et al. (2020) in their taphonomic experiments on fish found that the extreme environmental conditions in the Green River depositional environment could reduce decay and promote mineralization. Understanding the role of microbes and microbial by products in a range of fossilization conditions is therefore imperative and can be unpinned via a range of micrographical, mineralogical and molecular tools as discussed below.

### Morphological analysis of real concretion samples

This analysis provides a useful first screening to assess microbial mineral interactions, investigate the role of microbes and biofilms in different fossiliferous concretions and the physical nature of fossils. A range of microscopic and other microanalytical techniques have been utilized in the field of paleomicrobiology, including scanning electron microscopy, optical microscopy, fluorescence and confocal microscopy, atomic force microscopy and various analysis using synchrotron radiation.

Scanning electron microscopy (SEM) and petrographic analysis with coupled X-ray spectroscopy (SEM/EDS) reveals morphological features along with the chemical constituents, thereby providing a comprehensive picture of microenvironments during fossilization (Iniesto et al., 2013, 2016, 2017; Raff and Raff, 2014; Mähler et al., 2020; Dias and Carvalho, 2022). To date, a number of experimental studies on the direct influence of bacteria on mineralization and preservation have been conducted (in marine and non-marine settings) using different scanning electron micrographical and other elemental analysis tools (Briggs and Kear, 1993a,b,c; Hof and Briggs, 1997; Sagemann et al., 1999; Martin et al., 2005; Raff et al., 2008, 2014; Peterson et al., 2010; Butler et al., 2015; Naimark et al., 2016, 2018; Eagan et al., 2017; Mähler et al., 2020). In a recent study of insect fossils from the Lower Cretaceous Crato Formation *Lagerstätte* (part of the Santana Group of Brazil), SEM imaging revealed direct evidence of microbial morphotypes and textural features of mineralized EPS, thereby providing strong evidence of the influence of microbial mats in the fossilization process (Dias and Carvalho, 2022).

Optical (light) Microscopy helps in visualizing the fine details of an object by creating a magnified image through a series of glass lenses. Optical microscopy has been widely used in the study of concretions (e.g., Iniesto et al., 2015; Dias and Carvalho, 2022; Downen et al., 2022; Lin et al., 2023).

Confocal Scanning laser Microscopy (CSLM) creates sharp and distinctive images of an exact plane of a specimen (Relucenti et al., 2021). It allows the quantitative evaluation of structural parameters, thickness, and roughness. This technique also permits non-destructive analysis of the 3D architecture and 4D visualization of microfossils (Relucenti et al., 2021). This technique is widely used for the identification of bacteria and EPS distribution within the live biofilm matrix (Wagner et al., 2009; Reichhardt and Parsek, 2019). CSLM, in concert with various fluorescent stains, helps in understanding the different classes of macromolecules by calculating their abundance and distribution on biofilms in a very short period of time (Zhang et al., 1998; Hille et al., 2005; Wagner et al., 2009). However, CSLM has been applied and reported sparingly in fossil studies. Studies by Wagner et al. (2009) and Schopf and Kudryavtsev (2009) demonstrated that CSLM, when combined with Raman spectroscopy, enables observation of biofilms *in situ*, thus providing supplementary information about chemical properties and the distribution of components of EPS.

Fluorescence Microscopy is based on the emission of luminescence because of absorption of photons, when the samples treated with special fluorescent reagents exhibit Stokes shift. This technique can be used to identify structures in fixed and living biological samples (Lichtman and Conchello, 2005; Sanderson et al., 2014), and helps in understanding the viability of living and dead bacterial cells, biofilms, EPS and calcium carbonate precipitates (McMullan, 1995; Vernon-Parry, 2000). This technique has been recently utilized for investigation of internal architecture in steinkern spider fossils from Oligocene Aix-en-Provence Lagerstätte in southern France (Downen et al., 2022), which identified prolific microbial mat communities as likely being responsible for the preservation of infilled spider molds.

Atomic Force Microscopy (AFM) is a form of scanning probe microscopy, which uses a probe or tip to map the contours of the sample (Braga and Ricci, 1998). AFM is the measurement of the variation of force between the probe tip and the sample surface (Braga and Ricci, 1998) and thus helps in achieving a quantitative assessment of various interactive forces on biofilms under ambient conditions, or even on liquid surfaces, without any pre-treatment. This helps in generating a 3D image of surface topography. In general, these advantages make this emerging imaging technique useful for analyzing bacterial morphology, as well as their adhesive and elastic properties (Chatterjee et al., 2014).

Itrax X-Ray Fluorescence (XRF) core scanning extends traditional XRF characterization of the elemental composition of bulk sediment material by providing high-resolution elemental distributions. These have important implications for understanding paleoenvironmental and geochemical records (e.g., Croudace et al., 2006; Rothwell et al., 2006; Thomson et al., 2006), and can enable better understanding of the complex geochemical, redox and microbial conditions controlling carbonate fossilization (Croudace et al., 2006; Hunt et al., 2015; Hussain et al., 2020). The Itrax XRF core scanner simultaneously collects micro-XRF spectrometry elemental profiles, and optical and microradiographic images (Croudace et al., 2006). This provides an opportunity to study the micro-spatial relationship of elemental distributions within sample material. For example, detailed analyses of elemental distributions across carbonate concretions showed steep profiles of important elements (e.g., calcium) that were subsequently used to construct a diffusion model for concretion growth (Yoshida et al., 2015, 2018).

#### Omic techniques

Omic techniques are a set of high-throughput methods used to study the structure, function, and interactions of microbial communities in different environments. These techniques include DNA metabarcoding, metagenomics, metatranscriptomics, proteomics, and metabolomics, which analyze the genetic material, RNA transcripts, proteins, and metabolites present in a microbial community, respectively (see green pathways in Figure 7; Zarraonaindia et al., 2013; Shaffer et al., 2022). By using these approaches, researchers can gain insights into the diversity and complexity of microbial ecosystems and understand the roles of different microorganisms in these environments, their metabolic pathways, and their interactions with other organisms and their environment. Omic techniques have broad applications in microbiology, ecology, and biotechnology, and are helping to drive discoveries in fields such as environmental science and bioremediation (Garnatje et al., 2017; Gutleben et al., 2018; Zhang et al., 2018; Laczi et al., 2020; McElhinney et al., 2022). In recent years, there has been increasing interest in applying omic techniques to the study of fossils and the processes involved in their formation and preservation (Dong et al., 2019; Janssen et al., 2022). By integrating multiple omic technologies, it is possible to gain a more complete picture of the constituents and functions of microbial communities within concretions, and potentially unravel the mechanisms underlying concretion formation. For example, microbial biofilms have been shown to play a crucial role in the nucleation and growth of concretions (Frankel and Bazylinski, 2003; Grice et al., 2019; Janssen et al., 2022). It is possible to identify the predominant microorganisms and metabolic pathways involved in the formation of concretions by examining the structure of microbial communities and gene expression patterns in biofilms through techniques such as DNA metabarcoding, metagenomics, and metatranscriptomics (Babilonia et al., 2018; Onstott et al., 2019; Campbell et al., 2021). In addition, these techniques can also provide insights into the preservation of fossils within concretions. For instance, proteomic analysis can reveal the proteins and enzymes involved in the preservation of soft tissues in fossils (Boatman et al., 2019), while metabolomic analysis can identify the biochemical pathways responsible for the preservation of organic compounds (Janssen et al., 2022). The use of omic techniques in paleontology and sedimentology is a relatively recent development, and there is still much to be discovered regarding their potential and limitations. However, as the technology continues to improve, these techniques have the potential to revolutionize our understanding of fossil and concretion formation in modern settings.

#### Ancient DNA

The analysis of ancient DNA (aDNA) provides researchers with innovative ways to study the past (Yang and Watt, 2005). The study of ancient organisms is rapidly growing owing to recent methodological and technological advancement. It is now possible to obtain vast quantities of DNA data from ancient samples in a high-throughput manner and use this information to investigate the dynamics and evolution of past communities (Eisenhofer and Weyrich, 2019). For instance, by analyzing the DNA of microbial communities preserved in sedimentary layers, it is possible to reconstruct the diversity and abundance of past microbial assemblages and identify the environmental factors that influenced their evolution, as well as shed light on their role in mineral formation and biogeochemical cycling over geological time scales (Capo et al., 2022; Dong et al., 2022). The use of aDNA could potentially provide a window into the microorganisms existing at the time of concretion formation and allow for the identification of the original source organism. However, there are several limitations and challenges associated with aDNA analysis, such as the degradation of DNA over time and the risk of contamination from modern sources. Despite these challenges, in the presence of favorable preservation conditions, the application of aDNA analysis holds great potential as a tool for investigating concretions.

### Microbiology techniques

*Microbial culturing, enzymatic analysis, physico-chemical parameters and viability analysis* – Microbial culturing and enrichment is a simple and efficient method to enhance the multiplication of microbes (associated with fossil/sediment samples) by supplementation of nutrients under controlled laboratory conditions (Brock, 1999; Chapin and Lauderdale, 2007; Lewis et al., 2021). This aids in understanding the growth pattern, density, enumeration (colony forming units/ml) and metabolic properties of the enriched cultures along with their concretion/carbonate formation capability (Dhami et al., 2013a, 2017; Murugan et al., 2021). When trying to grow and study a specific microorganism under laboratory conditions (Figure 8, Approach 2), it is important to use a culture medium that closely mimics their natural environment. This helps the microbial colony to grow to its optimum, and enables better understanding of its specific needs and genetic makeup. Such minimal nutrient media have been designed and utilized in previous studies on enrichment of microbes from natural environments (Henson et al., 2016; Rahman et al., 2020; Ramachandran et al., 2020; Rodrigues and de Carvalho, 2022). Artificial sea water (ASW) media is generally used as a substitute for natural seawater to cultivate marine microbes as the latter often suffers from supply issues and seasonal variability and quality (Berges et al., 2001; Henson et al., 2016). Recently, halophilic archaea were cultivated from surface-sterilized middle–upper Eocene rock salt from the Yunying salt mine, China and the key feature that helped in the longevity of these microbes was their ability to keep their genomic DNA intact (Jaakkola et al., 2014).

Microbial community analysis is also conducted via omics tools (discussed above in omics section) followed by characterization of EPS, biofilm properties and any extracellular enzymes (as urease, carbonic anhydrase) produced by the grown cultures as demonstrated in prior studies (Dupraz et al., 2009; Li et al., 2015; Dhami et al., 2016; Al Disi et al., 2019; Murugan et al., 2021). Microbial culture supernatant is also investigated for change in physico-chemical parameters by microbial metabolic activities via a range of elemental analysis tools such as atomic absorption spectroscopy (AAS), Inductively coupled plasma mass spectroscopy (ICP-MS) and Fourier-transform infrared spectroscopy (FT-IR) (Li et al., 2015; Liu R. et al., 2021; Liu X. et al., 2021; Murugan et al., 2021).

### *In situ* characterization of concretion sequestered microfossils

It is advantageous to screen concretion collections with *in situ* characterization techniques to identify those with the most promising impregnations (e.g., large, exceptional preservation) for more detailed analytical attention. Given the typically limited amounts of fossil sample available and their precious nature, a range of appropriate micro characterization methods are often applied prior to non-destructive chemical analysis.

Many of the micro-graphical techniques described in the previous concretion section (see: *Morphological, molecular and microbiological characterization of concretions*) can also be applied to the sequestered microfossils. Typically, concretions are opened so that the fossils can be accessed when exposed, but some techniques also allow *in situ* interrogation of the enclosed fossils. For instance, SEM imaging methods can provide ultrastructural information of fossilized tissues. However, SEM is limited by low electron penetration depth, meaning that it is often more effective on exposed structures, and especially on carefully prepared ultrathin (<100 nm) sections; one drawback, therefore is that these are representative of only a small fraction of the specimen. 3D images of embedded fossils can be achieved through focused ion beam (FIB)-SEM or by combining sequential milling with concurrent Energy Dispersive Spectroscopy (SEM EDS) where despite the destructive milling approach, nanoscale fossil structures can be successfully imaged to provide insights into their chemistry, ultrastructure, taphonomy and biogenicity (Wacey et al., 2012; Brasier et al., 2015).

CSLM and Raman Spectroscopy can also be used for non-intrusive and non-destructive study of the 3D structure and chemical composition of EPS and fossils (Schopf and Kudryavtsev, 2009; Wagner et al., 2009). Schopf et al. (2010) utilized optical microscopy in combination with CSLM and other interdisciplinary approaches to successfully distinguish between authentic microbial fossils and microscopic “look-alikes.”

Atomic Force Microscopy (AFM) is now widely used for fossil observations and imaging in paleontological research (Benítez et al., 2019). AFM studies by Kempe et al. (2002, 2005) on organic-walled fossils from the upper Neoproterozoic Chichkan Formation of Southern Kazakhstan revealed stacked arrays of 200 nm-sized angular platelets of polycyclic aromatic kerogen on their walls. On comparison with SEM, AFM images provided higher resolution and 3D information about the organization of carbon within the cell. AFM has also been used to measure biofilm thickness and the height and “roughness” of EPS (Relucenti et al., 2021), and to identify the crystal structures of biogenic marine calcite and aragonite from fossil corals (Coronado et al., 2015), echinoderms (Stolarski et al., 2009) and mollusks (Casella et al., 2018; Benítez et al., 2019).

Computed tomography (CT) extends the centuries long use of X-Rays to help visualization of geological fossils (Hohenstein, 2004). X-ray computed tomography (XCT) scanners can successfully visualize fossils in un- or partially prepared concretions and has revolutionized the science of paleontology [see Sutton et al. (2014) for an excellent overview]. Applications of XCT to fossils in concretions include the anatomical description of a Devonian shark from Morocco (Klug et al., 2023); the analysis of an ichthyosaur skull from the Triassic of Svalbard (Roberts et al., 2022); and the visualization of trace fossils in driftwood from the Cretaceous–Paleogene Boundary in the USA (Maisch and Becker, 2022). XCT does have its limitations, however, including digital separation of fossils or matrix – in which case other techniques might be more effective (e.g., neutron tomography; see below).

Micro-CT (μCT) scanning, or high resolution XCT, is also now being popularly applied in paleontology. Recent concretion fossil studies include the reinterpretation of a purported Cambrian jellyfish fossil as a pseudofossil (Nolan et al., 2023); the visualization of the musculature and reproductive, digestive, and circulatory systems of a Devonian arthropod (Laville et al., 2023); the revelation in exquisite detail of the anatomy of Carboniferous millipedes from France (Lheritier et al., 2023); and the characterization of a Jurassic pseudoplanktonic community on a fossilized log (Little et al., 2023).

Synchrotron exploration of the X-ray and matter interactions on a range of geological materials can provide insights on morphology, elemental composition, oxidation states, crystalline structure, magnetic properties, and others, which can measurably contribute to the investigation of biogenicity of putative biosignatures (Callefo et al., 2019). Gueriau et al. (2020) utilized synchrotron radiation to generate X-ray fluorescence elemental maps of fossils representative of different taxonomic groups (arthropods, sarcopterygians and actinopterygians), types of preservation (compressed and three-dimensional fossils, including the ones with extensive soft-tissue mineralization), geological ages and depositional environments. Mineralogical maps were also generated in transmission geometry using a two-dimensional area detector placed behind the fossil.

Synchrotron propagation phase contrast micro-CT (PP-SRμCT/PPC SRμCT) identifies and maps mineral phases and their distribution at the microscale over centimeter-sized areas. Elemental information can be collected synchronously, informing on texture (preferential orientation), crystallite size and local strain. The extremely high resolutions (<100 nm; Vorontsov et al., 2023) achievable with PP-SRμCT has possibly led to the greatest paleontological advances when applied to concretions (Sanchez et al., 2012). This sophisticated technique has been applied to a variety of concretion fossils in recent years, including Devonian placoderm fishes from Australia (Trinajstic et al., 2022b), Triassic coprolites from Poland (Qvarnström et al., 2019a,b) and Jurassic cephalopods from France (Rowe et al., 2022). The non-invasive extraction of 3D information from homogeneously dense specimens has proved critical to some paleobiological studies (Tafforeau et al., 2006; Cunningham et al., 2014; Maldanis et al., 2016), revealing even the preservation of soft tissues.

#### Neutron scattering

Neutron tomography (NT) is similar to CT in as much as it enables digital separation of fossils from rocks (Bevitt, 2018). Advantageously, the contrast and penetration that can be achieved with neutrons is greater than with X-rays: X-rays interact with electrons surrounding an atom, whereas neutrons interact with atomic nuclei (Bevitt, 2018). NT has been used to visualize a variety of fossils in concretions, including Devonian fishes from Australia (Trinajstic et al., 2022b), a Jurassic ammonite from the United Kingdom (Cherns et al., 2022), a Cretaceous fish from Brazil (Pugliesi et al., 2019), a Cretaceous crocodyliform (with part of an ornithopod dinosaur in its body cavity) from Australia (White et al., 2022), a Cretaceous seed cone from New Zealand (Mays et al., 2017), and an Eocene plant from Antarctica (Dawson et al., 2014).

### Organic geochemical (and related) analysis of fossil OM

The isolation of the fossil from its concretion host is critical to the success of organic geochemical analysis of fossil OM. Separation is often achieved through physical methods, although these might not fully isolate the fossil and matrix. As a result, pure matrix is commonly analyzed separately from the fossil sample to avoid contamination and accurately determine the contributions of each component.

### Sample screening with high throughput bulk analysis

Bulk geochemical and key elemental analysis are common preliminary methods used to chemically screen concretions and their organic fossils. These methods often involve measuring the total organic and inorganic carbon contents of sediments, along with other elements commonly found in organic compounds such as nitrogen, sulphur and oxygen. Elemental analyses can be used to measure these elements in decarbonated sediments treated with hydrochloric acid, providing a total for each element.

Rock-Eval pyrolysis is a widely used method in determining the thermal stability and quality of OM (Espitalié et al., 1977). It measures the thermal evolution of carbon compounds over laboratory applied temperatures (i.e., 300 to 650°C), and can also provide an indication of the carbon, hydrogen and oxygen content of sediments.

### Molecular analysis by GC–MS

Natural OM (NOM) samples are typically subject to various wet chemistry and other preparation techniques to provide specific fractions appropriate for separate gas chromatography mass spectrometry (GC–MS) analysis. The most important technique for organic speciation – i.e., identification of organic compounds including biomarkers – in complex samples such as the NOM that can occur in concretions is GC–MS (see Grice and Eiserbeck, 2014 for a review).

#### Aliphatic and aromatic hydrocarbon speciation

The non-polar moiety of NOM is typically isolated in a solvent extractable or bitumen fraction which can be further resolved by liquid chromatography into saturate and aromatic fractions. These are then analyzed using GC–MS. The compounds detected in NOM samples can include biomarkers that act as molecular fossils of biological compounds like membrane lipids and pigments. After the deposition of organic matter, lipids and other compounds can be preserved and provide insights into past microbial activity (Brocks and Summons, 2014; Grice and Eiserbeck, 2014; Naeher et al., 2022). Molecular fossils can also include (metallo)porphyrins and proteins. These somewhat “functionalized” molecules are also relatively stable and often well-preserved in the rock record. Biomarkers when detected in NOM can provide a link to lipids and pigments of modern organisms (i.e., eukaryotes, bacteria, and archaea; Peters and Moldowan, 1992). In benign settings (e.g., concretions) hydrocarbon biomarkers may remain stable for hundreds of millions of years. Their detection in concretion fossils can therefore provide valuable taxonomic and environmental information about ancient ecosystems (see below).

#### Analytical pyrolysis of kerogen

The non-solvent soluble or kerogen fraction of NOM can also be interrogated by GC–MS following appropriate thermal (or chemical) treatments. Thermal energy can be used to break macromolecular OM into smaller products which can pass through GC columns, in a process referred to as analytical pyrolysis. An important advantage of kerogen analyses is that its covalently bound lattice is less vulnerable to overprinting from migrating hydrocarbons or other autochthonous inputs than the free hydrocarbon fraction (i.e., bitumen Peters and Moldowan, 1992).

Various pyrolysis devices have been developed for NOM kerogen analysis, but hydropyrolysis (HyPy) is now widely considered the best practice method because of its high detection sensitivity including of ancient biochemical signatures sequestered in inorganic substrates (Reinhardt et al., 2019). This pyrolysis event is conducted in a hydrogen-rich atmosphere to provide hydrogen donors that quench the reactivity of unstable ion and radical pyrolysates. This helps to maintain primary pyrolysate integrity often including the preservation of biomarker stereochemistry (Meredith et al., 2015). The HyPy released fraction is operationally trapped on a bed of silica gel, allowing subsequent isolation of saturate and aromatic hydrocarbon fractions which can be separately analyzed by GC–MS (or GC-isotope ratio-MS for compound specific isotope analysis).

Polar compound speciation and S-biogeochemistry: The polar fraction of solvent extracted (or HyPy liberated) NOM can be isolated by LC elution with polar solvents. Labile biomolecules, like proteins, lipids and sugars, can be in part sequestered through oxidative cross-linking, yielding N-, O-, and S- containing heterocyclic polymers (e.g., Wiemann et al., 2018a, 2020; McCoy et al., 2020). In particular an abundance of sulfides from microbial sulfate reduction (i.e., in low reactive iron/pyrite settings) can aid organic sulfurization during diagenesis, producing organic sulfur-rich macromolecular aggregates which can stabilize and preserve lipid biomarkers (Sinninghe Damsté and de Leeuw, 1990; Schouten et al., 1993; Aizenshtat et al., 1995).

The three-dimensional macromolecules produced by organic sulfurization are not amenable to GC–MS without further treatment. Raney Nickel is commonly used to selectively cleave the carbon-sulfur bonds of polar fractions, releasing a sulfur-bound organic fraction that can be analyzed by GC–MS (Sinninghe Damsté and de Leeuw, 1990). This approach has been widely used to study sulfurized lipids of Phanerozoic sediments. Heteroatomic organic compounds released by this process (or present in other fractions) may not be directly detectable by GC–MS without derivatization of specific chemical functionalities, e.g., acylation or silylation of acid or hydroxyl groups, respectively (Drozd, 1981). Some functionalized and high MW organic species can be detected directly by LC–MS, which can be advantageous for the analysis of thermally unstable or non-volatile species but is limited by increased band broadening effects compared to gas chromatography (Skoog et al., 2018).

#### Multidimensional GC–MS

Thanks to continued development, optimization and sophistication, GC–MS is being applied at increasing sensitivity and resolution. Both MS and GC detection can now be extended to multiple dimensions to support very high-resolution product detection. Two-dimensional gas chromatography (GC × GC) uses two different columns to separate compounds that co-elute in one-dimensional separations, greatly expanding resolution of complex mixtures (Scarlett et al., 2019). This allows high resolution physical separation which can aid the characterization of particularly complex mixtures. Tandem mass spectrometry (e.g., MS–MS) or Multiple Reaction Monitoring (MRM) can provide particularly selective analysis of target compounds such as molecular biomarkers (Mei et al., 2018). This upward technological trajectory has helped advance our knowledge of the biogeochemical pathways involved in the formation of biomarkers from natural product precursors during eogenesis (in the water column) and diagenesis (sediments and sediment water interface) and the unique palaeoenvironmental conditions required for their exceptional preservation (Grice et al., 2019).

### 2D surface analysis of NOM by particle bombardment techniques

The ability to correlate organic molecules with spatial and mineralogical sample features is an important consideration when interpreting exceptionally preserved fossils. Detailed molecular biomarker analysis, which typically requires destruction of bulk sample material, would ideally be complemented by spatially informative surface sensitive analytical methods.

Inductively Coupled Plasma Mass Spectrometry (ICP-MS) analysis using a laser probe can be used to create detailed maps of the elements present on the surface of a given sample. For example, Fe and S biochemical gradients measured across in concretion hosting an ichthyosaur fossil (Lower Jurassic Sachrang Formation, Germany) identified much lower microbial activity in the inner part of the concretion, which likely contributed to the excellent fossil preservation (Plet et al., 2016).

Time of Flight-Secondary Ion Mass Spectrometry (ToF-SIMS) is a technique that can analyze organic and inorganic molecules simultaneously with high mass and lateral resolution. Cluster primary ion beams improve the identification of high MW organic compounds with less analyte fragmentation. This technology is useful in various fields such as material science, biology, and surface analysis (Kollmer, 2004; Touboul et al., 2005). Important organic ToF-SIMS studies have included multiple biomarker investigations (e.g., Steele et al., 2001; Toporski et al., 2002; Siljeström et al., 2009, 2010, 2013, 2017, 2022; Thiel and Sjövall, 2011; Greenwalt et al., 2013; Thiel et al., 2014; Gren et al., 2017; Schweitzer et al., 2018). Organic ToF-SIMS analysis of geological and paleontological samples can yield mass spectral data from rare and precious specimens, altering only the uppermost few nanometers of analysis areas (Greenwalt et al., 2013; Lindgren et al., 2018, 2019; Baumgartner et al., 2023). As it employs no analyte separation (such as that provided by GC), it is not capable of resolving stereoisomers, and the isotopic composition of large organic molecules can be difficult to interpret. ToF-SIMS can complement traditional biomarker techniques by spatially correlating trace biomarker detection with organic sources, mineral phases, and structural features.

Raman spectroscopy is a non-destructive analytical method which can be used for chemical characterization of a wide range of sample materials. The incident source is a laser with a wavelength significantly different from the absorption wavelengths of the target analytes. The spectra produced are characteristic to each molecule, representing the energy difference between excitation and emission wavelengths of Raman active bonds. Use of Raman spectroscopy for *in situ* analysis of fossil specimens can identify preserved molecular functional groups, alongside intermolecular and organo-mineral relationships (Pelletier, 1999), making it a powerful tool for studying preserved organic matter and methods of biomineralization. Recent developments have given rise to widespread applications of Raman spectroscopy for analysis of biomolecular fossilization products (e.g., Yang et al., 2018; Wiemann et al., 2018a,b, 2019, 2020; Boatman et al., 2019; Fabbri et al., 2020; Norell et al., 2020; Steele et al., 2020).

### Stable isotope analysis

Analysis of the stable isotopic composition of sedimentary OM can help to distinguish a wide range of source inputs (Hedges and Keil, 1995; Grice and Brocks, 2011). In the case of fossiliferous concretions, these inputs include bacteria involved in concretion formation, the surrounding depositional environment, and the fossilized organism itself (Melendez et al., 2013b; Plet et al., 2017). Stable isotopic composition is controlled by three major factors: the isotopic composition of source elements, the isotopic fractionation during biosynthesis, and isotopic fractionations during burial and diagenesis (Hayes, 2001; Sessions, 2016).

Stable carbon and oxygen measurements of carbonate have helped to classify the source of concretion carbon, e.g., sulfate (or iron) reduction vs. methanogenic CO_2_ (Mozley and Burns, 1993; Pearson and Nelson, 2005; Cotroneo et al., 2016), or biogenic vs. abiogenic carbonate (Yoshida et al., 2015). The *δ*^34^S of pyrite gives information on the sulfur cycle during concretion formation. Enriched *δ*^34^S measurements of pyrite from Mazon Creek concretions suggest formation in sulfate-limited conditions and near total depletion of local dissolved sulfate by SRB during concretion formation (Cotroneo et al., 2016), whereas strongly depleted pyrite in Oligocene Boom Clay concretions (Belgium) indicates abundant dissolved sulfate (De Craen et al., 1999). Stable carbon isotope ratios of bulk organic matter (*δ*^13^C_org_) can be measured after removal of carbonate by reaction with dilute acid. Similarities in *δ*^13^C_org_ of Upper Cretaceous Holz Shale concretions (California) and host sediment was interpreted as evidence that OM was not strongly degraded during concretion formation (Loyd, 2017). Tripp et al. (2022) compared *δ*^13^C_org_ with compound-specific *δ*^13^C values of organic compounds from a Mazon Creek concretion (Illinois) preserving a coprolite (see below).

Compound-specific isotope analysis (CSIA) allows the stable isotope analysis of individual compounds in complex mixtures of sedimentary OM (Grice and Brocks, 2011). The technique was first developed for carbon (*δ*^13^C) and nitrogen (*δ*^15^N) (Matthews and Hayes, 1978), and has since been applied to hydrogen (*δ*^2^H, commonly written as *δ*D) (Burgoyne and Hayes, 1998) and sulfur (*δ*^34^S) (Amrani et al., 2009). The technical requirements for reliable and reproducible measurements are sufficient peak height for accurate measurement of the minor isotopes, and typically also baseline separation of peaks to allow the entire peak to be integrated without interference (Sessions, 2006; N.B. not necessary for *δ*34S CSIA).

## Challenges and future directions

The investigation of mineral concretions and their capacity to sequester the organic remains of extant organisms is still a relatively new and immature science. The information that concretions can convey about ancient organisms and ecosystems is primarily derived from the detection of molecular biomarkers (or distinctive morphological features) from the well-preserved fossils sequestered within them (Figure 8, Approach 1). Prior to the end goal of biomarker analysis, however, a large number and variety of other analytical approaches can assist the selection and interrogation of concretions with promising fossil targets. Different analytical approaches that can help to exploit this largely untapped biochemical record range from (i) fundamental evaluation of concretion formation controls for a better understanding of the propensity and distribution of those likely to sequester and preserve ancient organisms (Figure 8, Approach 1); to (ii) *in situ* characterization and imaging techniques able to identify concretions with promising fossil targets for more detailed geochemical analyses; and (iii) detailed geochemical characterization of the microfossils themselves.

This review has outlined promising methodologies utilized in preliminary studies exploring the chemistry of concretion-encapsulated fossils. Despite early advances in the development and application of techniques that have helped define concretion formation processes and characterize their OM record — including at submicroscopic morphologies and biochemical composition levels — this research area is best considered to be at a developmental stage. There is still much to learn about the mechanisms and controls on microbially mediated concretions and their preservation of NOM under different paleoenvironmental conditions. To better exploit the biological and geological information that fossiliferous concretions present, a number of research challenges will need to be addressed:

1. More effective literary, analytical and application integration of complementary Earth Science disciplines involved in concretion research, including microbiology, molecular biology, organic and inorganic geochemistry, paleontology and mineralogy. For instance, the role of various biogeochemical factors in the fossilization process (including those that have previously been implicated) can be further elucidated through studies of modern mineralogy, microbes, genes, and surface physicochemistry. Detailed observation and understanding of these parameters and processes will enable a better understanding of the environmental settings that led to concretion formation in deep time.
2. Design and develop laboratory simulations and modeling technology to evaluate field-based inferences of fossilization formation mechanisms (Figure 8, Approach 2). These microbial–mineral related processes are complex and often difficult to unravel directly from microbial, mineral and fossil data from the field, but can be systematically evaluated in artificially controlled situations or predicted from intelligent system models. Wide field data collection, including samples from different locations and timescales, will be important to guide the direction of laboratory experiments (Figure 8). The integration of large field, simulated and modeled data sets will provide new insights about microbe–mineral interactions in different concretion-forming environments (e.g., marine and non-marine settings).
3. Successful identification and selection of concretions that host the most promising fossils (i.e., the largest and most morphologically intact) will require the development and application of new field tools. Current evaluations of impregnated fossils with traditional imaging and other methods remain challenging. Development of new and accessible methods able to support *in situ* interrogation of organismic fossils, both in the field and laboratory would be highly beneficial. Appropriate field application will conveniently help to lessen downstream analytical loads.
4. Molecular speciation of organic sediments involves sophisticated, time-consuming procedures, restricting timely analytical processing of samples. The analytical challenges are intensified when conducted on trace amounts of organic analyses (well-preserved fossils are often very small) where particularly meticulous protocols need to be implemented to distinguish sample from background signals. This can be exacerbated when baseline organic signals are high (NB. biologically rich paleo-environments can result in highly dispersed organic content), which is the case at several famous sites with fossiliferous concretions, including the Carboniferous deposits at Mazon Creek, Illinois (coincident with a Pennsylvanian coal seam), the Eocene Green River Formation, Utah (oil shale formation); and the Cambrian Alum Shale, Sweden (shale rich; also high uranium concentration contributing to the radiological alteration of OM). New physical separation techniques able to better isolate fossils will be highly beneficial.
5. Research scientists are not always the most effective communicators, either within or beyond their direct research communities. Various logistics (e.g., time, lack of training) can challenge the public communication of research outcomes and the engagement of scientists in outreach initiatives. Nevertheless, scientists have an obligation (especially if backed by public funding) to openly communicate and share the results of their work, particularly those which tangibly impact society. The analysis of ancient organisms that are exceptionally preserved within mineral concretions will provide new information about evolutionary processes on Earth. A higher resolution of environmental and biological paleo-dynamics will add valuable context to understanding contemporary environmental and Earth science (ESS) issues, including modern climate change. Paleoenvironmental reconstructions help climate scientists assess the resilience of our planet’s biosphere, as well as its capacity to cope with and regulate widely fluctuating climate conditions. They also enable climate scientists to more accurately predict the environmental and ecological consequences of present-day climate trajectories – some of which are at close to historical highs (e.g., atmospheric temperature and CO_2_ rises over the last 200 years). An intensifying community focus on climate change presents an opportunity to improve societal understanding, attitudes, and behavior toward the environment (Bradley et al., 1999; Ballantyne et al., 2001). Effective communication and educational leverage of Earth Science (e.g., concretionary fossil) and climate change research will help to raise the EES literacy of the general public, including students (the next generation of Earth Scientists) who often have soft subject perceptions about ESS due to greater curricula focus on traditional science and mathematics subjects (Burg, 2003; Dawson and Carson, 2013). A basic EES learning will help to balance climate change perceptions, avoiding unwarranted alarm, but also encourage support for the phasing out of harmful industrial activities as societies transition to more environmentally sustainable practices. Effective science outreach programs should incorporate a variety of approaches, including but not limited to digital content, in-person events, workshops, media appearances, school visits, partnered programs and citizen science.

## Author contributions

ND, PG, SP, and KG wrote the manuscript as the leading authors. KG provided Figures 1, 8. MT and KG prepared Figures 5, 6 with input from other co-authors. AE prepared Figures 2, 7 with input from co-authors. AE and SP prepared Figure 3 and Table 1 with input from co-authors. HV and ND provided Figure 4 with input from co-authors. Table 2 was prepared by AE, PG, AH and KG along with other co-authors. All authors contributed to the article and approved the submitted version.
